# Genotype-to-Phenotype Associations in the Aggressive Variant Prostate Cancer Molecular Profile (AVPC-m) Components

**DOI:** 10.3390/cancers14133233

**Published:** 2022-06-30

**Authors:** Rama Soundararajan, Paul Viscuse, Patrick Pilie, Jingjing Liu, Souzana Logotheti, Caddie Laberiano Fernández, Daniele Lorenzini, Anh Hoang, Wei Lu, Luisa Maren Solis Soto, Ignacio I. Wistuba, Mingchu Xu, Xingzhi Song, Peter D. A. Shepherd, Nora M. Navone, Rebecca S. S. Tidwell, Guillermina Lozano, Christopher Logothetis, Jianhua Zhang, James P. Long, Marcos R. Estecio, Vasiliki Tzelepi, Ana M. Aparicio

**Affiliations:** 1 Department of Translational Molecular Pathology, The University of Texas MD Anderson Cancer Center, Houston, TX 77030, USA; cdlaberiano@mdanderson.org (C.L.F.); wlu3@mdanderson.org (W.L.); lmsolis@mdanderson.org (L.M.S.S.); iiwistuba@mdanderson.org (I.I.W.); 2 Department of Genitourinary Medical Oncology, The University of Texas MD Anderson Cancer Center, Houston, TX 77030, USA; pviscuse@mdanderson.org (P.V.); pgpilie@mdanderson.org (P.P.); slogotheti@upnet.gr (S.L.); anhoang@mdanderson.org (A.H.); pshepherd@mdanderson.org (P.D.A.S.); nnavone@mdanderson.org (N.M.N.); clogothe@mdanderson.org (C.L.); 3 Department of Genomic Medicine, The University of Texas MD Anderson Cancer Center, Houston, TX 77054, USA; jliu21@mdanderson.org (J.L.); mxu.china@gmail.com (M.X.); xsong3@mdanderson.org (X.S.); jzhang22@mdanderson.org (J.Z.); 4 Department of Pathology and Laboratory Medicine, Fondazione IRCCS Instituto Nazionale dei Tumori, 20133 Milan, Italy; daniele.lorenzini@istitutotumori.mi.it; 5 Department of Biostatistics, The University of Texas MD Anderson Cancer Center, Houston, TX 77230, USA; rsslack@mdanderson.org (R.S.S.T.); jplong@mdanderson.org (J.P.L.); 6 Department of Genetics, The University of Texas MD Anderson Cancer Center, Houston, TX 77030, USA; gglozano@mdanderson.org; 7 Department of Epigenetics and Molecular Carcinogenesis, The University of Texas MD Anderson Cancer Center, Houston, TX 77030, USA; mestecio@mdanderson.org; 8 Department of Pathology, University of Patras, 26504 Patras, Greece; btzelepi@upatras.gr

**Keywords:** AVPC-m, molecular heterogeneity, TP53, RB1, PTEN

## Abstract

**Simple Summary:**

We treat prostate cancer like it is one disease, but it is clear that it behaves like different diseases in different patients, with some having excellent responses to hormonal therapies and prolonged survivals, and others having virulent, aggressive courses and short survivals. These differences reflect different tumor biologies and therapeutic sensitivities, but the absence of molecular markers that identify each of these subsets makes it difficult to develop treatments specific to each. We found that alterations in two or more of the tumor suppressors TP53, RB1 and PTEN characterize the more virulent prostate cancers, and that patients with this molecular profile (called the aggressive variant prostate cancer molecular profile) appear to benefit more from combination chemotherapies than those without. The alterations in these markers can be determined either by staining tumor tissues or by examining their DNA. In this study, we used 28 mouse models of the human disease to assess the performance of various assays in determining these alterations. We found that although both staining and DNA sequencing are complementary, staining (which is a broadly available technique) is likely sufficient to make these determinations. Our results will inform the use of this molecular signature in clinical research and clinical practice.

**Abstract:**

The aggressive variant prostate cancer molecular profile (AVPC-m), composed of combined defects in TP53, RB1 and PTEN, characterizes a subset of prostate cancers linked to androgen indifference and platinum sensitivity. To contribute to the optimization of the AVPC-m assessment for inclusion in prospective clinical trials, we investigated the status of the AVPC-m components in 28 patient tumor-derived xenografts (PDXs) developed at MDACC. We subjected single formalin-fixed, paraffin-embedded (FFPE) blocks from each PDX to immunohistochemistry (IHC), targeted next-generation genomic sequencing (NGS) and Clariom-S Affymetrix human microarray expression profiling. Standard validated IHC assays and a 10% labeling index cutoff resulted in high reproducibility across three separate laboratories and three independent readers for all tumor suppressors, as well as strong correlations with loss-of-function transcriptional scores (LOF-TS). Adding intensity assessment to labeling indices strengthened the association between IHC results and LOF-TS for TP53 and RB1, but not for PTEN. For TP53, genomic alterations determined by NGS had slightly higher agreement scores with LOF-TS than aberrant IHC, while for RB1 and PTEN, NGS and IHC determinations resulted in similar agreement scores with LOF-TS. Nonetheless, our results indicate that the AVPC-m components can be assessed reproducibly by IHC using various widely available standardized assays.

## 1. Introduction

Androgen-indifferent prostate cancers are estimated to represent 20–30% of the lethal disease, but they still have limited therapeutic options and a dismal prognosis. This is in part due to a lack of biomarkers distinguishing them from the more common androgen-driven disease, thus preventing the development of therapies specific to them. In order to provide a framework that enables the development of effective therapies, the aggressive variant prostate cancers (AVPCs) have been defined as a subset of the disease with atypical and virulent clinicopathological features frequently associated with androgen-indifferent tumors (AVPC clinicopathological, AVPC-c) [1], which are also characterized by a molecular profile of combined defects in any two or more of the three tumor suppressors (TSPs) TP53, RB1 and PTEN (AVPC molecular, AVPC-m) [2]. In preclinical models, combined losses of *TP53* with *RB1* and/or *PTEN* have been associated with lineage plasticity and androgen indifference [3,4]. In addition, a randomized phase II study of cabazitaxel plus or minus carboplatin indicated that the AVPC-m could predict for benefit from the platinum combination [5]. These data support the hypothesis that the AVPCs should be distinguished from the typical androgen-driven prostate cancers as a unique subset of prostate cancers of therapeutic relevance.

However, the best way to identify the AVPC-m in clinical patient samples, whether by immunohistochemistry (IHC) and/or next-generation sequencing (NGS), remains to be determined. Indeed, in the clinical trial of cabazitaxel plus or minus carboplatin, the AVPC-m was determined by IHC and NGS of cell-free DNA, but concordance between these two assays was low [5]. Moreover, while preclinical models have modeled deletions of the tumor suppressors, clinical samples reveal a variety of genomic alterations in the AVPC-m components, including mutations, particularly in *TP53*. Different genomic alterations within one gene can lead to distinct phenotypes as shown, for example, for *FOXA1* [6]. We therefore set out to examine, in depth, correlations between protein expression (IHC), RNA expression (microarray) and DNA sequence (targeted sequencing) status of TP53, RB1 and PTEN, using a set of 28 patient tumor-derived xenograft (PDX) models, in an attempt to refine the definition of the AVPC-m and facilitate its implementation in clinical practice.

## 2. Materials and Methods

### 2.1. Patient Tumor-Derived Xenografts (PDXs)

**Figure 1:**
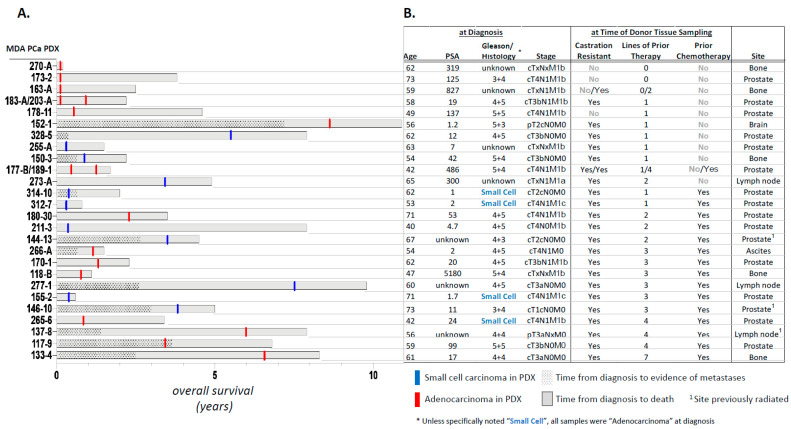
The 28 patient-derived xenograft (PDX) models of human prostate cancer used in this study. (**A**). Clinical course of the tumor tissue donors represented as a function of overall survival, along with morphology at the time of donor tissue retrieval (adenocarcinoma vs. small cell carcinoma). Also shown is time from diagnosis to evidence of metastasis. (**B**). Shown are age at diagnosis, PSA, Gleason score and tumor stage (as available), castration resistance, information on prior therapy and site of donor tissue for the 28 PDXs.

One formalin-fixed, paraffin-embedded (FFPE) block from each of 28 patient-derived xenograft (PDX) models of human prostate cancer was obtained from the MD Anderson Cancer Center (MDACC) Prostate Cancer PDX Program of ~200 models (MDA PCa PDX) [7]. Generation and propagation of these tumors as subcutaneous grafts in SCID male mice were performed as previously described [7,8] and approved by the MDACC ethics committee (PA12-0482). Please refer to Section 3.1 and Figure 1 for details on the patient cohort. The models were chosen to reflect the heterogeneity that is seen in the clinic (heterogeneity with respect to histology, tumor grading, prior lines of therapy and tumor site).

### 2.2. Immunohistochemistry

Serial 5-µm sections from the FFPE blocks were employed for hematoxylin-eosin (H&E) staining, as well as for IHC staining of the three tumor suppressor proteins, TP53, RB1 and PTEN. H&E staining was used to determine the morphology (adenocarcinoma vs. small cell) and % malignant cells in each of the 28 PDX tumor samples (Table S1). IHC was performed (using independently standardized lab protocols) in the CLIA-certified MDACC Clinical Pathology Laboratory (Lab 1), and in two additional research IHC laboratories at MDACC: one in the Department of Translational Molecular Pathology (Lab 2) and another in the Department of Genitourinary Medical Oncology (Lab 3). Details of antibodies used, experimental conditions employed, and positive and negative controls used in these analyses are shown in Tables S2 and S3. Epitopes recognized by the various antibodies are schematically depicted in Figure S1. Pathological evaluation of slides was performed by 3 independent pathologists (including a practicing clinical pathologist) using standard microscopy. Each sample therefore had 9 reads (3 labs X 3 pathologist readers). Pathologists did not have access to disease classification/group allocation information. The percentage of malignant cells with nuclear (RB1 and TP53) or cytoplasmic (PTEN) staining was recorded using the following intensity levels: 0 (no staining), 1+ (weak staining), 2+ (moderate staining), and 3+ (strong staining). In accordance with the previous definition [2,5,9], we considered the following to be aberrant: ≥10% of tumor cell nuclei positive for TP53; ≤10% tumor cell nuclei positive for RB1; and ≤10% of tumor cell cytoplasm positive for PTEN. These cutoffs are based on previously published studies in preclinical models and in samples obtained from participants in prospective clinical trials showing an association with outcomes and benefit from platinum-based chemotherapy [2,5,9]. Increased TP53 nuclear accumulation is considered an excellent surrogate marker for TP53 missense mutation status and is strongly associated with poor clinical outcomes for abiraterone/enzalutamide [10].

### 2.3. Genomic Analyses (T200 Targeted Sequencing Panel)

Second, additional serial sections from the same FFPE blocks from each of the 28 PDX models were subjected to genomic DNA extraction at the Biospecimen Recovery Facility at MDACC, using the QIAamp DNA FFPE Tissue kit from Qiagen (Catalog # 56404). DNA was RNase-treated during the purification process, and its quantity/purity measured using a Nanodrop spectrophotometer. DNA was submitted to the MDACC Advanced Technology Genomics Core for targeted sequencing (T200.1) of the exons of 201 cancer-related genes, including *TP53*, *RB1* and *PTEN* [11]. DNA was sequenced on an Illumina NovaSeq 6000 following the defined protocol for paired-end reads of 150 BP to a targeted depth of 200×. Twenty of the 28 samples yielded evaluable results. Sequencing reads were then aligned to the reference genome (human Hg19) using BWA mem [12] with 31 BP seed length after filtering out reads that aligned to the mouse genome (mm10). The aligned BAM files were subjected to mark duplication, re-alignment and re-calibration using Picard and GATK [11], before any downstream analyses. Somatic mutations were called using MuTect [13], and indels were called using Pindel [14]. Extractions from pooled normal human blood samples were sequenced using the same panel and used as a common reference for mutation and indel calling. Somatic mutations/indels supported by at least 20 reads in the PDX samples and 10 reads in the normal samples were included in the downstream analyses. Further filtering of mutations/indels was done by excluding events that have been reported in normal human populations, unless identified as potentially pathogenic by COSMIC. An R package Maftools [13] was used to process the downstream mutation analysis. DNA copy number variations were determined using an in-house application ExomeLyzer [15], followed by CBS [16] segmentation. The CNTools Bioconductor package (https://bioconductor.org/packages/release/bioc/html/CNTools.html, accessed on 24 April 2020) was used for further downstream analyses. Copy number variations with log2 ratios ≤ −0.8 were defined as deletion. Variations with log2 ratios ≥ 0.8 were defined as amplification. Gene mutations with allele frequencies > 0.1 were considered significant. Additionally, the data were filtered for germline mutations. Those events reported in the snp129 database (for germlines) were removed, except for those also reported in the cosmic72conf database (composed of somatic mutations). For clinical significance, those with CADD scores (Combined Annotation Dependent Depletion) (https://cadd.gs.washington.edu/) calculated in the pipeline were defined as follows: “benign” for scores of <10, “pathogenic” for scores of ≥20, and “VUS (variation of unclear clinical significance)” for scores between 10 and 20. Those with no CADD scores were defined as follows: “benign” for 3′ or 5′ UTR or flank, silent and noncoding; “pathogenic” for nonsense, frameshift or splice; and “VUS” for others.

### 2.4. Gene Expression Analysis (Clariom-S Microarray)

Total RNA was extracted from an additional 2 FFPE sections from each of the 28 PDX models at the MDACC Biospecimen Recovery Facility, using the High Pure FFPET RNA Isolation Kit from Roche (Catalog # 06650775001). RNA quantity and purity were measured using a Nanodrop spectrophotometer and reconfirmed using Qubit. The RNA was DNase-treated and subjected to whole transcriptome expression profiling using the Clariom-S Affymetrix human microarray platform, at the Advanced Technology Genomics Core. All 28 samples yielded evaluable results. The Clariom-S array has 56 transcript cluster probes for TP53, 77 probes for RB1, and 8 probes for PTEN. A transcript cluster, here, refers to a group of one or more probes covering a region of the genome representing all exonic transcription known for that region and corresponding to a particular gene. This array assays over 20,000 well-annotated genes and covers over 337,100 transcripts in the human genome. Arrays were scanned with the Affymetrix scanner and quantified into CEL files with image and signal intensities. The CEL files were analyzed by R Bioconductor packages “oligo” and “pd.clariom.s.human”.

### 2.5. Statistical Analyses

To determine the agreement among the three reviewers calling TSP loss using immunohistochemical data alone, we used Fleiss’ kappa, a measure of observer agreement for categorical data [17]. Fleiss’ kappa values generally range between 0 and 1, with 0 indicating low/no agreement and 1 indicating perfect agreement. The following categories were used to interpret the agreement scores [17]: 0 = poor agreement, 0.01–0.20 = slight agreement, 0.21–0.40 = fair agreement, 0.41–0.60 = moderate agreement, 0.61–0.80 = substantial agreement and 0.81–1.00 = almost perfect agreement.

To determine associations of tumor suppressor loss as judged by IHC or T200 genomic analyses, with functional tumor suppressor pathway loss, normalized measures of loss-of-function transcriptional scores were dichotomized with numbers >0 classified as “loss” and ≤0 classified as “not loss”. This dichotomized loss was tabulated with IHC, single nucleotide variations (SNV), copy number variations (CNV) or combination (yes vs. no) for each relevant marker. Cohen’s kappa, a measure of agreement, was calculated with 95% confidence intervals using SAS 9.4 (SAS Institute Inc., Cary, NC, USA). A kappa value of 1 means perfect agreement and 0 means perfect disagreement [18].

## 3. Results

### 3.1. Clinical Course of PDX Donors

The 28 MDA PCa PDXs were obtained from 26 patient donors (Figure 1). MDA PCa PDXs 117-9, 118-B, 133-4, 144-13, 146-10, 150-3, 152-1, 155-2, 163-A, 170-1, 173-2, 177-B, 178-11, 180-30, 183-A and 189-1 have been previously reported [2,7,8,19]. Details of the donors’ clinical course are described in Supplementary Data S1. Their median age at diagnosis was 59.5 (range 40–73) years. Four (15.4%) of the 26 donors with known histology at diagnosis had primary small cell carcinomas, and 8 (30.8%) of the 26 were found to have small cell morphology during the castration-resistant progression of the disease. Of the 28 PDXs, sixteen (57.1%) were obtained from the prostate, 5 (17.8%) from bone, 3 (10.7%) from lymph nodes, 1 (3.6%) from the brain and 1 (3.6%) from ascitic fluid. MDA PCa 265-6 was derived from a patient diagnosed with primary small cell carcinoma but, following chemotherapy, his prostate tumor yielded a PDX with adenocarcinoma morphology. In contrast, MDA PCa 163-A had adenocarcinoma morphology, but its donor developed small cell carcinoma 6 months after the PDX was obtained. In all, 11 (39.3%) of the 28 PDXs had small cell morphology. Four (14.3%) were derived from tumors that were not castration-resistant, 7 (25.0%) had been exposed only to androgen deprivation therapy (commonly leuprolide), and 16 (57.1%) had been exposed to ≥1 chemotherapy regimens (range 1–7). Two patients donated two PDX lines at different times during their course. MDA PCa 183-A and MDA PCa 203-A were derived from bone marrow biopsies obtained from the donor at diagnosis and at the time of castration resistance, respectively. Both were adenocarcinomas. MDA PCa 177-B and MDA PCa 189-1 were derived from the donor’s prostate tumor before and after chemotherapy, respectively. Interestingly, the former was an androgen receptor (AR)-negative poorly differentiated carcinoma with morphology suggestive of neuroendocrine features, while the latter was an AR-positive adenocarcinoma. These PDXs represent the spectrum of histological grades, metastatic sites and lines of treatment encountered in clinical practice.

### 3.2. Assessment of TSP Defects by IHC

**Figure 2:**
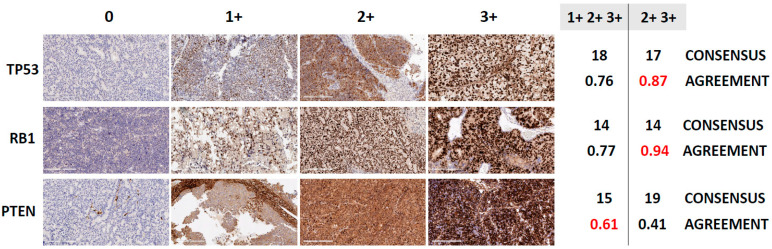
Consensus and agreement calling in immunohistochemistry (IHC)-based determination of tumor suppressor loss, in 28 PDX models of human prostate cancer. Figure 2 shows representative photomicrographs of PDXs stained for TP53, RB1 and PTEN demonstrating different staining intensity levels, and the proportion of samples a reviewer votes with the consensus vote for different markers (TP53, RB1 and PTEN) and definitions of positive (1+2+3+ or 2+3+ IHC % labeling indices). The consensus vote is the majority determination of abnormal for that sample across the 9 reviews (3 labs × 3 reviewers). Shown are the number of abnormal samples based on the consensus of the reviews and the Fleiss κ, which measures the level of agreement among the reviewers. Fleiss κ values of 0.41–0.60 indicate moderate agreement, 0.61–0.80 substantial agreement, and 0.81–1.00 almost perfect agreement. Also shown are representative IHC images reflecting 0, 1+, 2+ and 3+ staining intensities for TP53, RB1 and PTEN (magnification: 20×, scale bar = 200 µm).

For each of the tumor suppressors that compose the AVPC-m, serial sections from each of the PDXs were subjected to immunohistochemical analyses in three independent laboratories, using three different antibodies and epitope retrieval methods. Thus, nine reads (3 laboratories × 3 readers) were obtained for each one (shown in Figure S2) and scored either negative (0) or positive with 1+, 2+ or 3+ staining intensities. As stated above, we considered the following to be aberrant: ≥10% of tumor cell nuclei positive for TP53; ≤10% of tumor cell nuclei positive for RB1; and ≤10% of tumor cell cytoplasm positive for PTEN [2,5]. We then asked what the variance in the calls (normal vs. abnormal) was amongst the 9 reads per sample by determining the *consensus* (defined as the majority opinion of normal vs. abnormal for a given sample) and the *agreement* (frequency at which the three reviewers make the same call of normal vs. abnormal for a given sample, quantified using Fleiss’ kappa, Figure 2). Of note, the agreement (kappa values) amongst reviewers was higher for p53 and RB1 than for PTEN. For TP53 and RB1, the % of cells with ≥2+3+ intensity reads resulted in higher kappa values than the 1+2+3+ reads, although the number of normal vs. abnormal calls was nearly the same for TP53 and the same for RB1. For PTEN, 1+2+3+ intensity reads resulted in higher agreement among labs/readers. Overall, although there were some outliers, the majority of the IHC interpretations were aligned across laboratories and readers.

### 3.3. Correlation between TSP Loss-of-Function Transcriptional Scores and IHC Results

Next, we calculated loss-of-function transcriptional scores for each of the TSPs using previously published methods [20] and loss-of-function signatures generated in patient tumor samples [21,22,23]. All except the one for TP53 had been developed using prostate cancer samples. The TP53 loss-of-function signature table (Supplementary Table S2 and Figure 2 in [21]) provides a total of 20,501 genes, with *p*-values up to 1, absolute coefficient down to 0, and Weight.up/Weight.dn values down to 0. A meaningful and functional list of signature genes was sub-selected from among these 20,501 genes, considering the gene weight and signature size. Those with a Weight.up value ≥0.9, and a Weight.down value ≥0.9 were selected. This resulted in the top 280 most-weighted up/down-regulated genes, the length of which is manageable and comparable with some frequently used gene signatures such as in GSEA (Gene Set Enrichment Analyses). The RB1 and PTEN loss-of-function signatures were employed as published without sub-selection.

**Figure 3:**
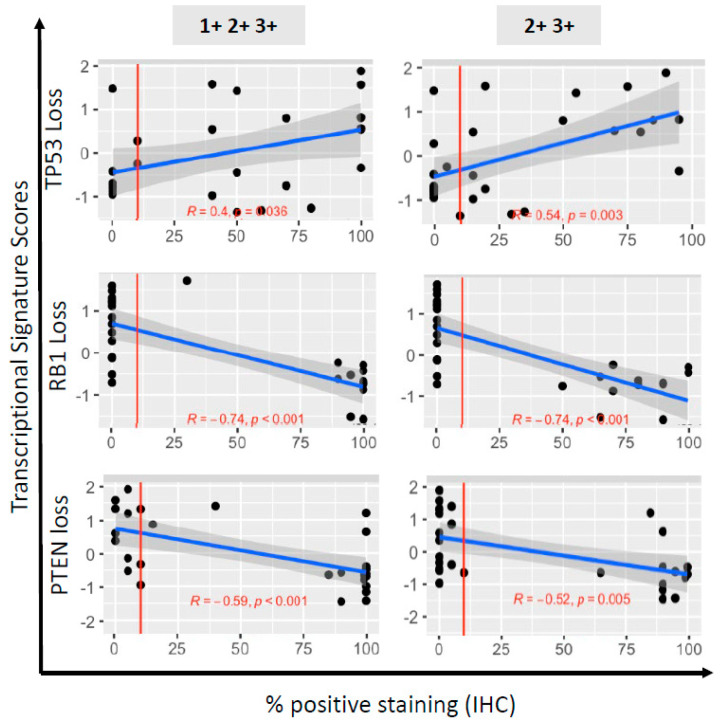
Correlation of immunohistochemistry (IHC)-based determination of TSP loss with functional downstream tumor suppressor pathway loss, in 28 PDX models of human prostate cancer. Shown are scatterplots of marker transcriptional scores (TP53, RB1 or PTEN pathway loss) against IHC % labeling indices. The 3 loss scores (rows) are regressed on percentage positive by IHC either 1+2+3+ (left column) or 2+3+ (right column). The red vertical line at 10% corresponds to the threshold for determining abnormal or normal based on IHC staining. The blue line is the ordinary least squares regression fit. The grey shaded region around each line is a 95% confidence interval set for the fit. The correlation (R) and significance of correlation (*p*-value for testing if correlation is 0) are included in each plot in red text.

Correlations between IHC labeling indices (using % tumor cells with 1+2+3+ vs. 2+3+ intensities) and transcriptional scores were overall strong, and similar amongst readers and laboratories (Figure S3). Shown in Figure 3 are reads obtained in Lab 1 (the CLIA-certified MDACC Clinical Pathology Laboratory) and scored by the practicing clinical prostate cancer pathologist, as referenced. In most cases, the points are clustered near 0% or 100% IHC staining, indicating that changing the 10% labeling index threshold would not have a large effect on the categorization of the samples as normal or abnormal.

For both TP53 and RB1, the correlation between the transcriptional score reflecting their loss-of-function was more significant for the labeling indices based on 2+3+ intensities than for those based on 1+2+3+ intensities. As stated above, the inter-reader/protocol agreement (Figure 2) was also greater for the labeling indices based on 2+3+ intensities for both tumor suppressors. Thus, we used the labeling indices based on 2+3+ intensities to categorize the PDX as abnormal for TP53 (*n* = 17, 60.7%) and RB1 (*n* = 14, 50.0%) by IHC. In contrast, for PTEN, the correlation between the transcriptional scores reflecting PTEN pathway loss and the labeling indices, was stronger for 1+2+3+, compared with 2+3+ intensities, as was the inter-reader/protocol agreement. Therefore, we used the labeling indices based on 1+2+3+ staining intensity to determine abnormal PTEN status (*n* = 15, 53.5%). Of note, of those that retained PTEN cytoplasmic staining, 8/13 (61.5%) samples had lost nuclear staining, which has been associated with more aggressive cancers [24]. Overall, the correlations between IHC and loss-of-function transcriptional scores were in the expected direction for all three tumor suppressors, and were improved when the intensity of the stains was taken into account.

### 3.4. Assessment of TSP Defects by NGS

**Figure 4:**
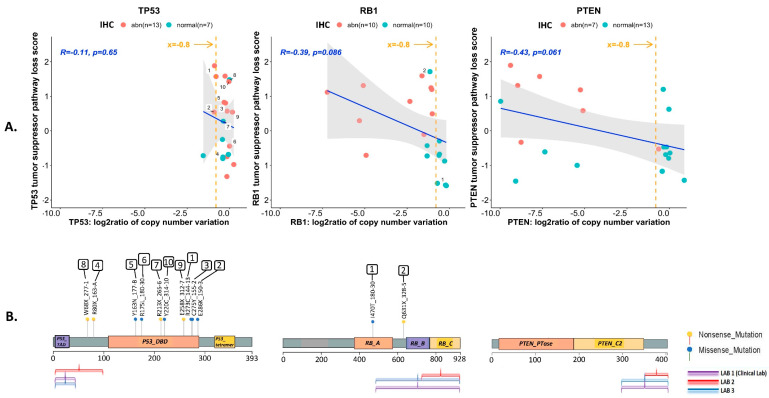
Genotype-to-phenotype correlations for each of the three tumor suppressors (TP53, RB1 and PTEN) using 20 MDA PCa PDX samples. (**A**) Correlations between the log2 ratios of the copy number variations for each of the tumor suppressors (TP53, RB1 and PTEN) identified by T200 analyses, and the transcriptional scores for tumor suppressor pathway loss. The orange line indicates the cutoff used to infer deep deletion (x = −0.8). The blue line is the ordinary least squares regression fit. The grey shaded region around each line is a 95% confidence interval set for the fit. The correlation (R) and significance of correlation (*p*-value for testing if correlation is 0) are included in each plot in blue text. Also shown is the categorization of each sample as normal (green dot) or abnormal (red dot) based on immunohistochemical (IHC) analyses. Samples harboring single nucleotide variations (mutations) are numbered. (**B**) Location of the identified mutations on the primary protein structure of the corresponding TSP, and the epitopes recognized by antibodies used for IHC staining. Nonsense mutations are shown as yellow dots, and missense mutations are shown as green dots. No mutations were identified for PTEN. Key functional domains are highlighted for p53 protein (trans-activation domain (*TAD*), DNA binding domain (*DBD*) and the oligomerization domain (*tetramer*)), RB1 protein (small pocket region containing the A (*RB_A*) and B (*RB_B*) domains and the C-terminal domain (*RB_C*)), and PTEN protein (phosphatase domain (*PTEN_PTase*) and C2 domain (*PTEN_C2*)).

Twenty (71.4%) of the 28 PDXs yielded DNA of sufficient quantity and quality for targeted sequencing analysis. For *TP53*, 4 (20%) had deletions (144-13, 146-10, 133-4, 170-1), 6 (30%) had missense mutations in the DNA binding domain, and 4 (20%) had truncating nonsense mutation (2 in the DNA binding domain, and 2 in the linker region preceding the DNA binding domain). All of the PDXs with *TP53* missense mutations had labeling indices ≥10%. One of the PDXs with a nonsense mutation in the DNA binding domain had a labeling index of 15%. The remainder of the PDXs with nonsense mutations had labeling indices of 0. For RB1, 13 (65%) had deletions, 1 (5%) had a missense mutation and 1 (5%) had a nonsense mutation. For PTEN, 10 (50%) had deletions but no mutations were detected. The overall correlations between the log2 ratios of the copy number variations and the loss-of-function transcriptional scores for each of the tumor suppressors are shown in Figure 4, along with the location of mutations on the protein structure, and the epitopes recognized by antibodies used for IHC staining. Log2 ratios of the copy number losses were significantly lower for *PTEN* and *RB1* than for *TP53*. Considering all pathogenic genomic alterations, 13 (65%), 14 (70%) and 9 (45%) of the 20 PDXs were categorized as genomically abnormal for *TP53, RB1* and *PTEN,* respectively (Table S4). Shown in Figure S4 are the tripartite relationships (IHC–transcriptomics–genomics) for all three TSPs across the PDXs.

Next, we investigated the value of adding the NGS results to the IHC determination of TSP abnormality. For this, we calculated the agreement between IHC calls and inclusion of single nucleotide variations (SNV) and/or copy number variations (CNV) (where available) with the loss-of-function transcriptional scores for each of the TSPs, using Cohen’s kappa (Table 1). Although the sample size is small, our data suggest that for *TP53*, the agreement between the IHC results and its loss-of-function transcriptional score is weak (k = 0.381). While including SNV along with the IHC calls yielded a better k (k = 0.468), use of SNV calls alone yielded the best agreement with the loss-of-function transcriptional scores for TP53 (k = 0.500). For RB1, determination of abnormality by IHC alone yielded the highest agreement with its loss-of-function transcriptional score (k = 0.700). For PTEN, both the IHC and CNV calls had similar agreement levels (k = 0.468 and k = 0.490, respectively) with its loss-of-function transcriptional scores. When combining IHC and NGS results, 18 (90%), 14 (70%) and 10 (50%) of 20 PDXs were determined to be abnormal for TP53, RB1 and PTEN, respectively, with a total of 17/20 (85%) bearing the AVPC-m by IHC and/or NGS (AVPC-m^+^, Table S4). By these combined criteria, only 3/20 (15%) PDXs (117-9, 183-A and 203-A) were determined to be AVPC-m^−^ (lacking the AVPC-m), precluding any comparisons between AVPC-m^+^ and AVPC-m^−^ tumors.

These data indicate that both IHC and NGS are complementary but could be used in isolation for the assessment of these tumor suppressor defects if one or another is not available.

## 4. Discussion

The AVPC-m consisting of combined defects in any two of the three tumor suppressors TP53, RB1 and PTEN has been linked to distinct clinicopathological features, a poor prognosis, androgen indifference and platinum sensitivity in advanced prostate cancers [1,2,5]. These observations support the notion that the AVPC-m enriches for a distinct biological subset of the disease, and can serve to select patients for prospective clinical trials evaluating therapies specific to androgen-indifferent disease. Here, we evaluated the performance of standard IHC and NGS assays to determine aberrancies in the TSP components of the AVPC-m in a panel of FFPE samples from PDX models, in order to inform its clinical application. We show that standard validated IHC assays for each of the tumor suppressors yield similar results across laboratories, and that a 10% labeling index cutoff resulted in high reproducibility across readers and expected correlations with loss-of-function transcriptional scores (LOF-TS) for the three TSPs. Importantly, excluding 1+ intensity stains in the determination of abnormal TP53 and RB1 IHC calls resulted in greater inter-reader/protocol agreements and stronger correlations with their LOF-TFs.

The strengths of our study include the determination of TSP status by protein, RNA and DNA assessments in single FFPE blocks for each of the PDXs (limiting the potential confounding effect of heterogeneity across PDX models on the results and making comparisons across assays more robust) and the use of IHC and NGS assays that replicate those available in most clinical settings. Weaknesses include the relatively small number of models and the absence of correlation with clinical outcomes. Indeed, the PDX models used in this study were derived from patients at various stages of their disease and represent a heterogeneous population. Despite this, when taking both IHC and NGS results into account, the majority were AVPC-m^+^. This is in line with previous reports indicating that PDX models are biased towards more aggressive phenotypes [25].

Previous studies have analyzed the associations between IHC and genomic sequencing results for the three TSPs of interest in prostate cancer tissues [9,26,27,28,29]. In line with our results, these studies have shown that TP53 expression by IHC is strongly correlated with *TP53* missense mutation detection but, because prostate tumor tissues show low basal expression of TP53, IHC cannot detect p53 loss that results from nonsense/frameshift/indel alterations or copy number losses [9,26,27,28]. In addition, they show that both hemizygous and homozygous allelic RB1 and PTEN losses correlate with loss of protein expression, but also that both RB1 and PTEN protein expression can be lost despite absence of allelic loss [28,29]. It is likely that some of this discordance can be explained by structural variants and promoter methylation, which have been described for each of the tumor suppressors and associated with their inactivation in prostate cancer [30,31], but would not have been detected by the techniques used here. The advantage of IHC, of course, is that it is more widely available and turn-around times are significantly faster than for NGS, a factor that becomes important when treatment decisions need to be made for men with virulent prostate cancer.

In this study, we used loss-of-function transcriptional scores as the comparator for IHC and NGS results. Two caveats to this approach should be noted: first, the transcriptional scores are relative to each other, thus it is conceivable (although unlikely) that all 28 PDXs might have TP53, RB1 or PTEN functional deficiencies relative to other tumors. Second, these tumor suppressors interact with numerous proteins, are regulated by many different processes, and have myriad downstream functions that are often context-dependent [24,32,33,34]. It is possible that the loss-of-function transcriptional signatures may perform differently in different clinical settings, e.g., in hormone-naïve vs. castration-resistant prostate cancers, which will be evaluated in future studies.

## 5. Conclusions

The purpose of the AVPC framework is to enable the distinction of clinically meaningful, therapeutically relevant prostate cancer subsets, which can allow the development of therapies specific to them. Akin to other tumor classifiers (e.g., triple-negative breast cancer), there will surely be therapeutically relevant heterogeneity within the AVPC subset, and further refinement of this molecular signature is needed. Until then, for the purposes of ongoing translational and clinical research, while NGS adds value to the determination of TP53 status, our data indicate that IHC is a reliable assay to identify the TSP-LOF for AVPC molecular profiling. It is possible that substantially increasing the sample size for these analyses would determine that a mixed-methods approach would pan out to be the most beneficial (IHC for RB1 and PTEN, and NGS for TP53); however, it is pertinent to note that IHC-based determination of TSP aberrancy did indeed correlate with tumor suppressor pathway LOF. Altering how readers *call* normal/abnormal is critical for IHC to hold up. For RB1 and TP53, a 2+3+ intensity scoring agreed with functional TSP pathway loss, whereas for PTEN, a 1+2+3+ intensity scoring performed better. Ultimately, our observations need to be validated prospectively in patient samples, and we expect that the AVPC-m will be further refined with additional studies to increase its robustness and predictive value. However, the findings from this study serve to inform our analysis in samples from participants in our ongoing (e.g., NCT02703623, manuscript in preparation) and planned prospective clinical trials, representing a step in that direction.

## Figures and Tables

**Table 1:** Associations of immunohistochemical (IHC) and genetic measures of determining TSP aberrations with functional tumor suppressor pathway loss among 20 patient tumor-derived xenografts of human prostate cancer. Comparisons were made between IHC, copy number variations (CNV), single nucleotide variations (SNV) and combinations of the three, in terms of their ability to determine abnormal tumor suppressor function (true positives). Shown are the Cohen’s kappa measure of inter-method agreement with the 95% confidence limits.

TP53		Loss No	Loss Yes	Kappa (95% Confidence Interval)
		N	N	
**IHC Abnormal**				0.381 (−0.021, 0.784)
	No Yes	5 4	2 9	
**CNV**				0.048 (−0.309, 0.405)
	No Yes	7 2	8 3	
**SNV**				0.500 (0.122, 0.878)
	No Yes	7 2	3 8	
**CNV/SNV**				0.381 (−0.021, 0.784)
	No Yes	5 4	2 9	
**CNV/SNV/IHC**				0.239 (−0.056, 0.534)
	No Yes	2 7	0 11	
**SNV/IHC**				0.468 (0.126, 0.810)
	No Yes	4 5	0 11	
**RB1**				
**IHC Abnormal**				0.700 (0.389, 1.000)
	No Yes	9 2	1 8	
**CNV**				0.612 (0.298, 0.925)
	No Yes	7 4	0 9	
**SNV**				0.022 (−0.265, 0.309)
	No Yes	10 1	8 1	
**CNV/SNV**				0.519 (0.199, 0.839)
	No Yes	6 5	0 9	
**CNV/SNV/IHC**				0.519 (0.199, 0.839)
	No Yes	6 5	0 9	
**CNV/IHC**				0.612 (0.298, 0.925)
	No Yes	7 4	0 9	
**PTEN**				
**IHC Abnormal**				0.468 (0.071, 0.865)
	No Yes	10 2	3 5	
**CNV**				0.490 (0.106, 0.874)
	No Yes	9 3	2 6	
**CNV/IHC**				0.400 (0.006, 0.794)
	No Yes	8 4	2 6	

## Data Availability

Not applicable.

## Supplementary Materials

The following supporting information can be downloaded at: https://www.mdpi.com/article/10.3390/cancers14133233/s1. Figure S1: Epitopes recognized by various antibodies used for immunohistochemical staining of the three tumor suppressor proteins—TP53, RB1 and PTEN. Figure S2. % labeling indices of immunohistochemical staining of TP53 (nuclei), RB1 (nuclei) and PTEN (cytoplasms) across the 28 MDA PCa PDX models used in this study. Figure S3. Correlations of immunohistochemistry % labeling indices with tumor suppressor pathway loss for TP53, RB1 and PTEN. Figure S4. Tripartite correlations between tumor suppressor protein expression, downstream pathway function and genomic status for the three tumor suppressor proteins—TP53, RB1 and PTEN—across the 28 MDA PCa PDX models used in this study. Table S1. List of 28 MDA PCa patient tumor-derived xenograft models used in this study. Table S2. Details of antibodies used for immunohistochemical staining of TP53, RB1 and PTEN. Table S3. Positive and negative controls used for immunohistochemical staining of TP53, RB1 and PTEN tumor suppressor proteins. Table S4. Tabulation of tumor suppressor (TP53, RB1 and PTEN) loss in the 28 PDX models, determined by immunohistochemical staining as well as by T200 genomic analyses of copy number loss and pathogenic single nucleotide variations. Supplementary Data S1. Clinical history of the 26 MDA PCa PDX donors.

## Abbreviations

AVPC	aggressive variant prostate cancer
AVPC-m	AVPC molecular profile
TSP	tumor suppressor protein
PDX	patient tumor-derived xenograft
IHC	immunohistochemistry
FFPE	formalin-fixed, paraffin-embedded
LOF-TS	loss-of-function transcriptional score
NGS	next-generation sequencing
SNV	single nucleotide variation
CNV	copy number variation
