# Supplementary material for: Genotype-to-Phenotype Associations in the Aggressive Variant Prostate Cancer Molecular Profile (AVPC-m) Components
Source: Cancers (Basel). 2022 Jun 30;14(13):3233. doi: 10.3390/cancers14133233 (PMC9265062; doi:10.3390/cancers14133233)

Supplementary Figure S3

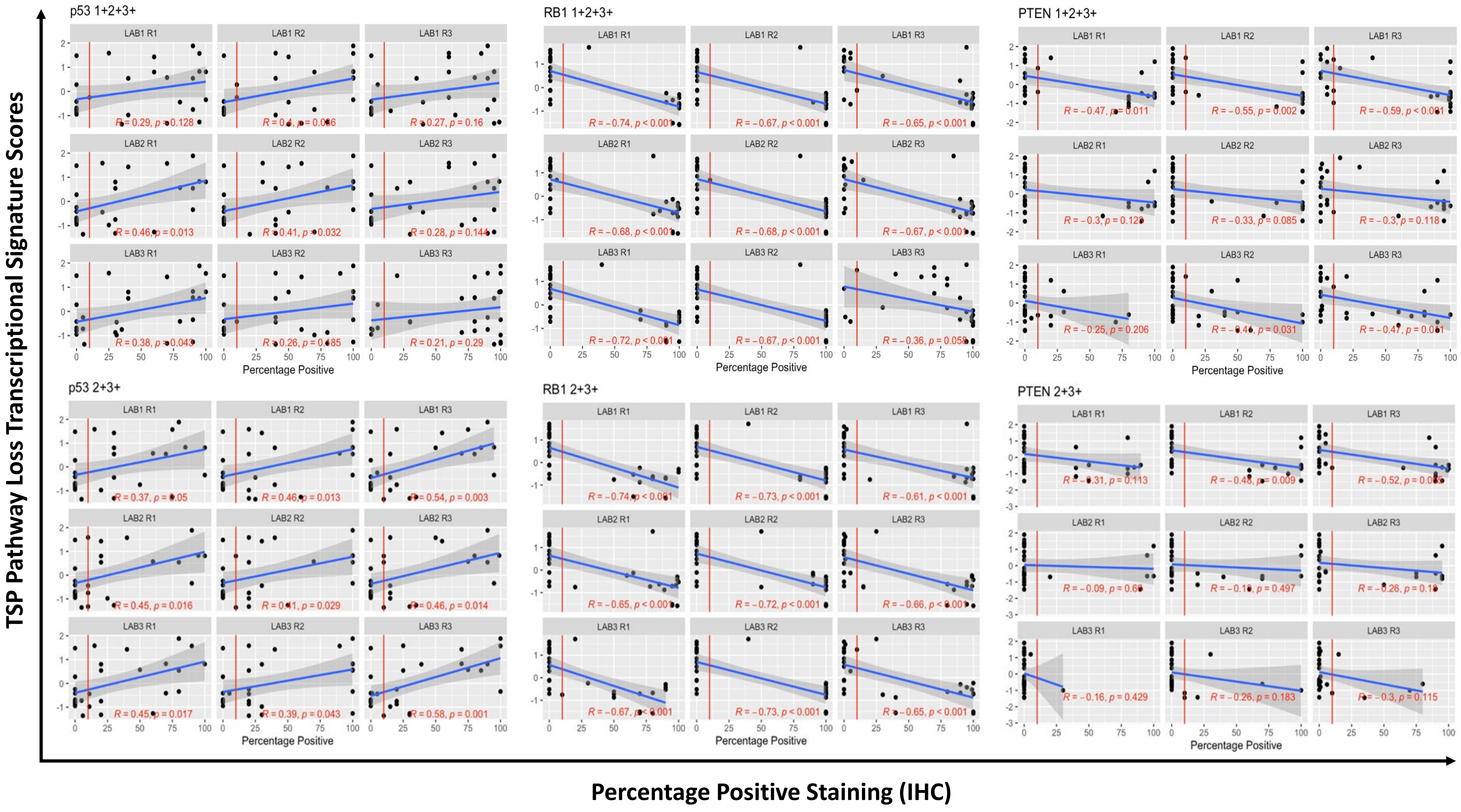

Supplement: Supplementary file 1 [file cancers-14-03233-s001.zip › Figure S3.pdf]
