# Supplementary material for: Genotype-to-Phenotype Associations in the Aggressive Variant Prostate Cancer Molecular Profile (AVPC-m) Components
Source: Cancers (Basel). 2022 Jun 30;14(13):3233. doi: 10.3390/cancers14133233 (PMC9265062; doi:10.3390/cancers14133233)
Supplement: Supplementary file 1 [file cancers-14-03233-s001.zip › Supplementary Files-Captions.pdf]

**Supplementary Files-Captions**

**Figure S1.** Epitopes recognized by various antibodies used for immunohistochemical staining of the three tumor suppressor proteins (TSPs) (TP53, RB1 and PTEN). Schematic depicting primary structure of the TSPs, along with key functional domains and antigenic regions recognized by antibodies used in the CLIA-certified clinical laboratory (Lab 1), and two other research laboratories (Lab 2 and Lab 3).

**Figure S2.** % labeling indices of immunohistochemical staining of TP53 (nuclei), RB1 (nuclei) and PTEN (cytoplasms) across the 28 MDA PCa PDX models used in this study. Shown are 1+2+3+ reads from all 3 laboratories (Lab 1, Lab 2 and Lab 3) and all 3 reviewers (R1, R2 and R3) (*i.e.,* 9 reads per sample). The ordering of the PDX models is from highest to least TSP abnormality, data sorted for TP53 first, RB1 next and PTEN last. The dotted horizontal line at 10% corresponds to the threshold cutoff for determining normal or abnormal.

**Figure S3.** Correlations of immunohistochemistry (IHC) % labeling indices with tumor suppressor pathway loss for TP53, RB1 and PTEN. Scatterplots of marker transcriptional scores (measuring TP53, RB1 and PTEN tumor suppressor pathway loss) against IHC % labeling indices, for each laboratory and reviewer (total of 3 laboratories (Lab 1, Lab 2, Lab 3) and 3 reviewers (R1, R2, R3)). The red vertical line at 10% corresponds to the threshold for determining abnormal or normal. The blue line is the ordinary least squares regression fit. The grey shaded region around each line is a 95% confidence interval set for the fit. The correlation (R) and significance of correlation (*p*-value for testing if correlation is 0) are included in each plot in red text.

**Figure S4.** Tripartite correlations between tumor suppressor protein expression, downstream pathway function and genomic status for the three tumor suppressor proteins—TP53, RB1 and PTEN—across the 28 MDA PCa PDX models used in this study. Shown are % labeling indices of IHC staining for p53 (2+3+ staining intensities, nuclei), RB1 (2+3+ staining intensities, nuclei) and PTEN (1+2+3+ staining intensities, cytoplasms). Included are reads from all 3 laboratories (Lab 1, Lab 2 and Lab 3) and all 3 reviewers (R1, R2 and R3) (*i.e.*, 9 reads per sample). The dotted horizontal line at 10% in the labeling index plots corresponds to the threshold cutoff for determining normal or abnormal. For each tumor suppressor protein, the heatmaps below the labeling index plot indicate protein expression status by IHC (normal vs. abnormal), relative downstream pathway function by TSP loss transcriptomic scoring using Clariom-S analyses, and copy number variation (CNV) status by T200 analyses ($\log 2 < -0.8$).

**Table S1.** List of 28 MDA PCa patient tumor-derived xenograft models used in this study. Included are information on morphology (adenocarcinoma (adenoCa) *vs.* small cell carcinoma (SCC)), % purity (% malignant cells) and representative H&E images of each PDX at 20x magnification.

**Table S2.** Details of antibodies used for immunohistochemical staining of TP53, RB1 and PTEN, along with the antigen-retrieval protocol used by each of the three laboratories employed in this study.

**Table S3.** Positive and negative controls used for immunohistochemical staining of TP53, RB1 and PTEN tumor suppressor proteins.

**Table S4.** Tabulation of tumor suppressor (TP53, RB1 and PTEN) loss in the 28 PDX models, determined by immunohistochemical staining as well as by T200 genomic analyses of copy number loss and pathogenic single nucleotide variations. Shown are final determinations of AVPC-m status for each PDX. AVPC-m: aggressive variant prostate cancer molecular profile.

**Supplementary Data S1.** Clinical history of the 26 MDA PCa PDX donors.
