# Supplementary material for: Genotype-to-Phenotype Associations in the Aggressive Variant Prostate Cancer Molecular Profile (AVPC-m) Components
Source: Cancers (Basel). 2022 Jun 30;14(13):3233. doi: 10.3390/cancers14133233 (PMC9265062; doi:10.3390/cancers14133233)
Supplement: Supplementary file 1 [file cancers-14-03233-s001.zip › SupplementaryData S1.pdf]

Supplementary Data S1: Clinical history of the 26 patient-derived xenograft tumor donors

MDA 177 and MDA 189. The patient donor of the MDA 177 and MDA 189 PDX presented with lower urinary symptoms and rectal pain at the age of 42. He was diagnosed in 2009 with a PSA 486, Gleason 5+4, cT4N1M1b *de novo* metastatic prostate cancer to bone and lymph nodes. His disease progressed within 6 months of starting bicalutamide and LHRH agonist treatment. At this time, a large tumor mass was palpable on digital rectal exam and a transrectal biopsy revealed a **poorly differentiated adenocarcinoma that was immunonegative for PSA, synaptophysin and chromogranin A.** **MDA 177** was derived from this biopsy (Fig.1A). He then underwent treatment with carboplatin-docetaxel, cisplatin-etoposide and paclitaxel-adriamycin-bevacizumab before undergoing a pelvic exenteration that yielded **MDA 189**. The pathological exam of the surgical specimen revealed a majority of tumor outside the prostate reported as a high-grade carcinoma that was immunonegative for PSA and AR, while positive for CD56. In contrast, residual tumor within the prostate was described as an **adenocarcinoma demonstrating some therapy effect, that was immunopositive for PSA and AR** (Fig.1B). This pattern, in which the invasive edge of the tumor is poorly differentiated and AR negative, while the tumor remaining in the prostate retains adenocarcinoma morphology and AR expression, has been previously described (1, 2). Tragically, the patient developed liver metastases one month later and succumbed to his disease in 11/2010, within 21 months of his diagnosis.

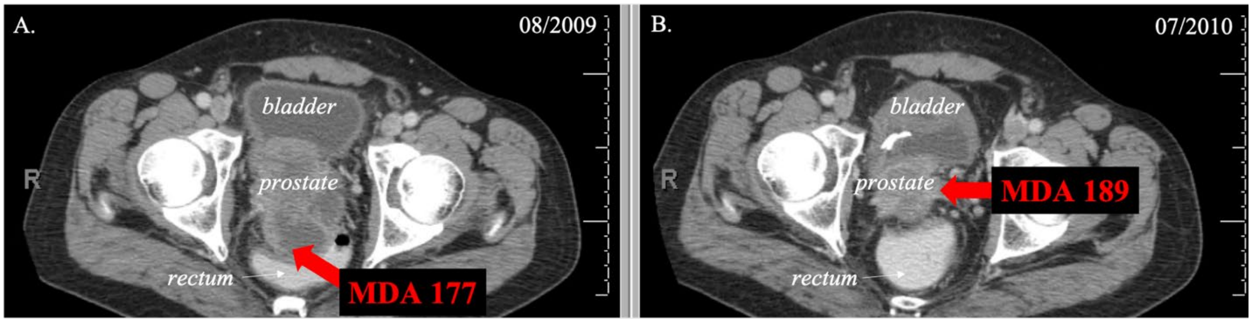

Figure 1A, 1B (CT scans)

<u>MDA 118.</u> The patient donor of the MDA 118 PDX presented with night sweats, malaise and decreased urinary stream. He was diagnosed in 10/2001 with a PSA 5,180, Gleason 5+4, cTxNxM1b *de novo* metastatic prostate cancer to bone at the age of 47. His PSA nadired at 0.6 but progressed approximately 6 months after starting combined androgen blockade. He progressed rapidly through a bicalutamide withdrawal and 'antineoplastons' plus trastuzumab therapy. A biopsy of a left buttock metastasis in 04/2002 revealed a poorly differentiated adenocarcinoma. He underwent radiation to right shoulder and clivus, followed by DES plus weekly docetaxel through which he progressed rapidly with new metastases to the liver. He was started on paclitaxel plus carboplatin to which he responded for approximately 4 months. He then developed increased pain in left hip and groin. In 10/2002 a fine needle aspiration of a left superior ramus metastasis revealed a **poorly differentiated carcinoma that was immunonegative for PSA, PAP, chromogranin A and synaptophysin**. MDA 118 was derived from this biopsy. He was treated with weekly adriamycin and oral etoposide, from which he derived brief symptom improvement, and was discharged to home hospice. He died in 12/2002, within 14 months of his diagnosis.

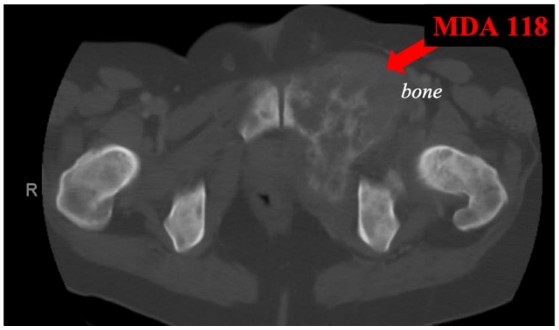

MDA 117-9. The patient donor for MDA 117-9 presented with urinary frequency and vague back discomfort. He was diagnosed in 03/1998 with a PSA 99, Gleason 5+5, cT3bN0M0 prostate cancer at the age of 59. He was treated with leuprolide, high dose bicalutamide, a dendritic vaccine and an EGFR tyrosine kinase inhibitor on clinical trials and docetaxel and estramustine followed by a salvage cystoprostatectomy in 06/2002 revealing a **prostatic adenocarcinoma** diffusely replacing prostate with invasion of bladder neck as well as pelvic and presacral lymph nodes with extranodal extension, and yielded **MDA 117-9**. As described by Tzelepi et al. both the donor tumor and the PDX were **immunopositive for AR, chromogranin A, synaptophysin and RB1** (3). He later developed diffuse bone metastases and extensive lymphadenopathy with increase in PSA to 973. He was treated with imatinib and docetaxel on clinical trial, and then ifosfamide and gemcitabine chemotherapy before succumbing to his disease in 05/2003, 5 years after his diagnosis.

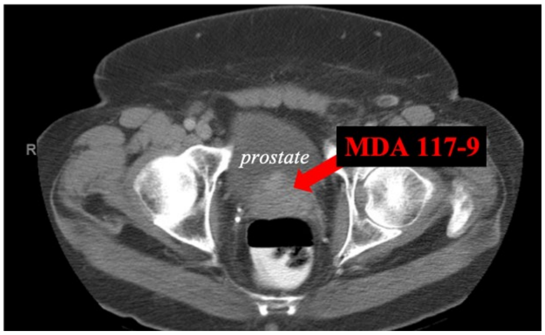

MDA 133-4. The patient donor for MDA 133-4 presented with an elevated PSA on routine examination. This led to a diagnosis of a PSA of 17, Gleason 4+4, cT3aN0M0 prostate cancer in 11/1996 when he was 61 years old. He was treated with concurrent radiation, leuprolide and bicalutamide. In the year 2000 he developed metastatic disease to the bone, resumed androgen deprivation therapy, and was later treated with palliative radiation to T8, and on clinical trials with paclitaxel-estramustine-thalidomide, docetaxel-imatinib and then ifosfamide-doxorubicin-gemcitabine, mitoxantrone, diethylstilbestrol and gefitinib. He developed a subcapital fracture of the right femoral neck requiring hemiarthroplasty in 01/2005. Pathology confirmed **metastatic carcinoma** consistent with known prostatic primary and yielded **MDA 133-4**. His course was complicated by sepsis and he passed away on home hospice in 06/2005, 9 years after his diagnosis.

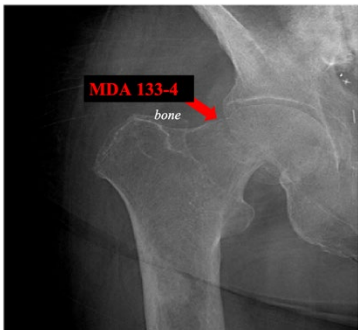

<u>MDA 137-B.</u> The patient donor for MDA 137-B was diagnosed with a PSA unknown, Gleason 4+4, pT3aNxM0 prostate cancer, in 06/1999 at the age of 56. He was treated with a prostatectomy but had a PSA recurrence and was treated with androgen deprivation therapy, diethylstilbestrol, and a vaccine-based therapy followed by an experimental angiogenesis inhibitor on clinical trials. In 2006 he was found to have a local recurrence with involvement of the left ureter and bladder neck. He was treated with docetaxel-carboplatin and radiation followed by a radical left nephroureterectomy with lymphadenectomy. Pathology revealed **metastatic prostatic adenocarcinoma** involving lymph nodes, ureter, renal pelvis and perinephric adipose tissue, and yielded **MDA 137-B**. He later developed bone metastases, was treated with a PDGF inhibitor on study, and palliative radiation to the left orbit, but succumbed to his disease in 05/2007, 8 years after his diagnosis.

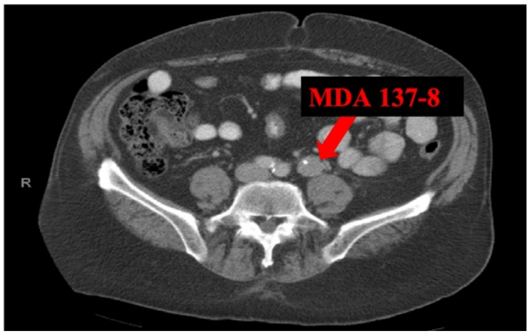

<u>MDA 144-13.</u> The course and clinicopathological features of the patient donor for MDA 144-13 have been previously described (1, 3). He was diagnosed with a PSA unknown, Gleason 4+3, cT2cN0M0 prostate cancer in 12/2002, at the age of 67. He was treated with radiation therapy and concurrent androgen deprivation therapy for 9 months. He had a PSA recurrence in 04/2006 and resumed androgen deprivation therapy. However, he had rapid development of bladder outlet obstruction and hematuria within 2 months. A cystoscopy revealed a nodular prostatic mass invading the bladder and a biopsy revealed **small cell carcinoma** which was negative for PSA and PAP but positive for CD56, chromogranin, and synaptophysin. He was treated on a clinical trial with docetaxel-carboplatin and salvage cisplatin-etoposide. He underwent a salvage cystoprostatectomy with pelvic lymph node dissection and sigmo-proctectomy with sigmoid colostomy in 05/2007 which revealed a **mixed small cell carcinoma and adenocarcinoma of the prostate**. The surgical specimens yielded 8 PDX sublines that have been previously described (1). MDA 144-13 was derived from a tumor fragment obtained from the bladder neck and was composed of small cell carcinoma. The patient recovered from surgery but ultimately succumbed to his disease in 09/2007, 5 years after his diagnosis.

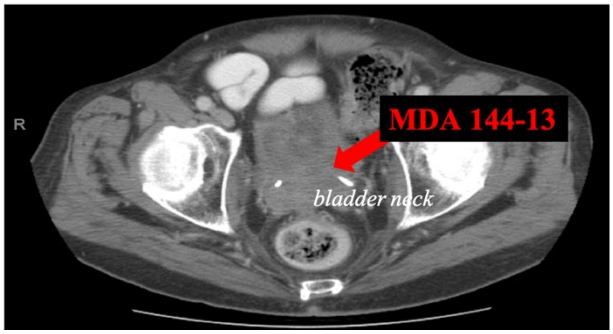

MDA 146-10. The patient donor for MDA 146-10 was diagnosed with a PSA 10.7, Gleason 3+4, cT1cN0M0 in 11/2002, at the age of 73, for which he was treated with brachytherapy in 01/2003. His PSA nadired at 1.9 in 08/2003 but subsequently rose to 75 in 08/2006 in conjunction with severe irritative and obstructive urinary symptoms, as well as rectal and penile pain, but absent distant metastatic disease. He initiated androgen deprivation therapy at that time but developed castration resistance by 12/2006 and bicalutamide was added to his treatment. In 05/2007 he underwent a cystoscopy with transurethral resection of tumor at the bladder neck. Pathology revealed **poorly differentiated neuroendocrine carcinoma, immunopositive for CK AE1/AE3, synaptophysin, chromogranin A, PSA and PAP** in 90%, 99%, 95%, 10% and 20% of neoplastic cells respectively, and immunonegative for CK903 and p63. He was treated with 2 cycles of docetaxel and then underwent a salvage cystoprostatectomy in 08/2007. The pathology of this sample was reported in (3), and consisted of a **mixed tumor containing adenocarcinoma and small cell carcinoma components**. The former stained positive for AR (90%) and PSA (20%) but was negative for chromogranin A and synaptophysin, while the latter was negative for AR and PSA, and positive for both neuroendocrine markers. **MDA 146-10** was derived from this specimen and was composed of small cell carcinoma. Unfortunately, he recurred in 11/2007 and passed away in 11/2008, 5 years after his diagnosis.

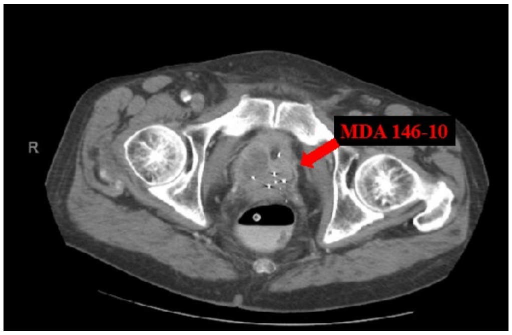

<u>MDA 150-3.</u> The patient donor for 150-3 was experiencing progressive fatigue and urinary tract symptoms, and was diagnosed with a PSA 42, Gleason 5+4, cT3bN0M0 in 11/2006 at the age of 54. He was treated on a clinical trial of leuprolide and temsirolimus and underwent a radical prostatectomy in 02/2007. Pathology revealed a **high-grade adenocarcinoma**. He recurred with a right occipital skull mass in 10/2007. In 12/2007 he underwent a right occipital craniotomy and resection of metastasis. Pathology revealed **metastatic high-grade carcinoma, negative for PSA and PAP but positive for chromogranin and synaptophysin** and yielded **MDA 150-3**. He was then treated with 5 cycles of etoposide and cisplatin and 1 cycle of etoposide and carboplatin completed in 05/2008. Restaging studies showed recurrent disease in his occipital bone and the liver. He underwent reopening and re-do of craniotomy and tumor resection in 07/2008. Pathology showed **small cell carcinoma, negative for PSA and PAP but positive for synaptophysin and CD56.** This was followed by whole brain and C-spine radiation in 08/2008 but in 09/2008 he presented with a cord compression requiring a complex spinal surgery including a T3 vertebrectomy which again revealed **high-grade carcinoma with neuroendocrine features.** This was followed by radiation to the thoracic spine and a course of low dose paclitaxel and dasatinib. He then went on hospice and succumbed to his disease in 02/2009, 2 years after his diagnosis.

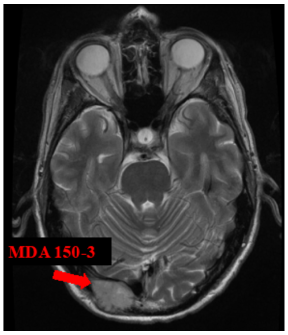

<u>MDA 152-1.</u> The patient donor for 152-1 was diagnosed with a PSA 1.2, Gleason 5+3, cT2cN0M0 prostate cancer in 05/1997, at the age of 56. He was treated with radical prostatectomy followed by radiation therapy and a total of 1 year of leuprolide achieving an undetectable PSA until 06/2005. His PSA rose slowly and in 06/2006 he was found to have a lung nodule which was biopsied and showed **metastatic carcinoma with positive staining for PSA and PAP and negative neuroendocrine marker expression**. This lesion grew and he resumed leuprolide in 12/2006. In 01/2008 he developed facial palsy and hemiparesis and underwent a craniotomy with resection of a right frontal lobe mass. Pathology revealed **prostatic ductal adenocarcinoma, PSA and PAP positive** and yielded **MDA 152-1**. He completed whole brain radiation in 02/2008. He was treated with 3 cycles of carboplatin and docetaxel but his performance status deteriorated and he went on hospice. He passed away in 10/2008, 10 years after his original diagnosis.

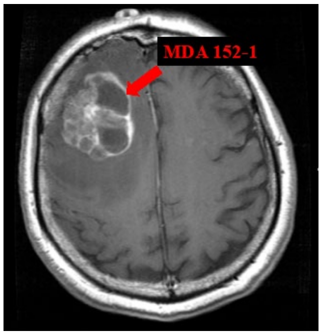

MDA 155-2. The patient donor for MDA 155-2 presented with lower urinary tract symptoms and severe pelvic pain and was diagnosed with a PSA 1.7, primary small cell carcinoma, cT4N1M1c de novo metastatic disease in 10/2007, at the age of 71. He progressed through 2 cycles of docetaxel and carboplatin but had a dramatic response to 4 cycles of cisplatin and etoposide. In 03/2008 he underwent a pelvic exenteration with the pathology report noting **small cell carcinoma**. This tumor was reported in (3), and consisted of pure small cell carcinoma, negative for AR and PSA, and positive for chromogranin A and synaptophysin. This surgical specimen yielded **MDA 155-2**. Unfortunately, he relapsed in the brain shortly thereafter, received whole brain radiation and transitioned to hospice. He passed away in 05/2008, 7 months after his diagnosis.

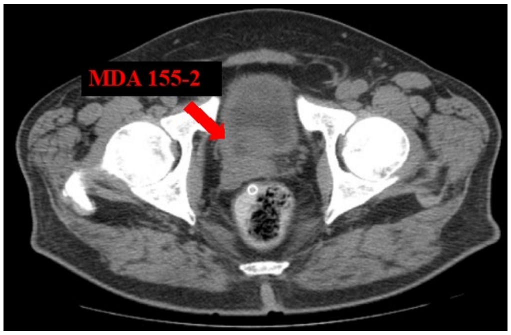

<u>MDA 163-A.</u> The patient donor for MDA 163-A was 49 years old when he presented with rib pain and lower urinary tract symptoms and was diagnosed with a PSA 136.9, cT4N1M1b, Gleason 5+5 de novo metastatic adenocarcinoma of the prostate in 10/2008. During his workup he developed a cord compression treated with high dose steroids and radiation, followed by leuprolide. In 12/2008 he remained symptomatic and repeat prostate biopsies revealed **high grade prostate adenocarcinoma**. Of note his testosterone at the time was 121ng/dL and PSA had increased to 182ng/dL. This tumor sample yielded **MDA 163-A**. He resumed leuprolide, his symptoms improved and his PSA nadired at 0.9ng/dL. However, in 07/2009 his obstructive symptoms returned and a repeat prostate biopsy showed **mixed adenocarcinoma and small cell carcinoma.** He was treated with multiple chemotherapy regimens, on and off clinical trials, but unfortunately passed away in 07/2011, 2.5 years after his diagnosis.

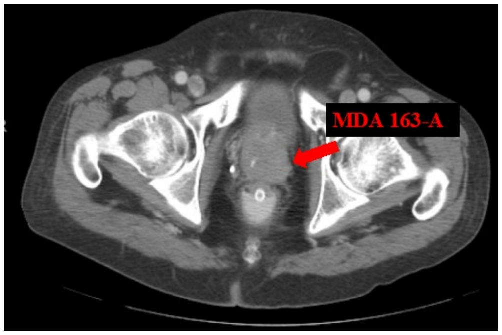

<u>MDA 170-1.</u> The patient donor for MDA 170-1 was diagnosed with a PSA 20, Gleason 4+5, cT3bN1M1b de novo metastatic prostate adenocarcinoma in 09/2007 at the age of 62. He was treated with leuprolide and docetaxel, followed by cyclophosphamide-vincristine-dexamethasone. In 04/2009 he underwent a cystoprostatectomy revealing a **prostate adenocarcinoma** that yielded **MDA 170-1**. He suffered numerous complications and was ultimately diagnosed with bony metastases for which he initiated docetaxel-carboplatin in 11/2009 but progressed rapidly and passed away from his disease in 02/2010, 2.3 years from his diagnosis.

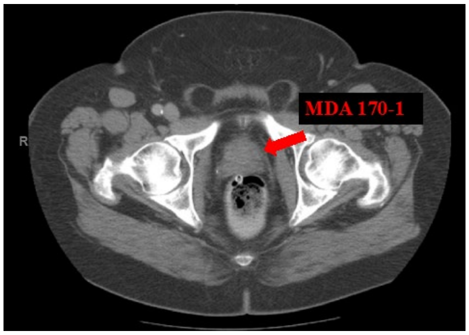

<u>MDA 173-2.</u> The patient donor for MDA 173-2 presented with urinary retention and gross hematuria and was diagnosed with a PSA 125, Gleason 3+4, cT4N1M1b de novo metastatic prostate cancer in 05/2009, at the age of 73. A biopsy of a lytic bone lesion in 05/2009 revealed metastatic adenocarcinoma and a TURP in 05/2009 revealed **prostatic adenocarcinoma**, Gleason 3+4. This tissue sample yielded **MDA 173-2**. He was treated with leuprolide, radiation to his thoracic spine, DES, docetaxel-dasatinib/placebo on clinical trial, abiraterone, cabazitaxel and enzalutamide before succumbing to his disease in 03/2013, 4 years after his diagnosis.

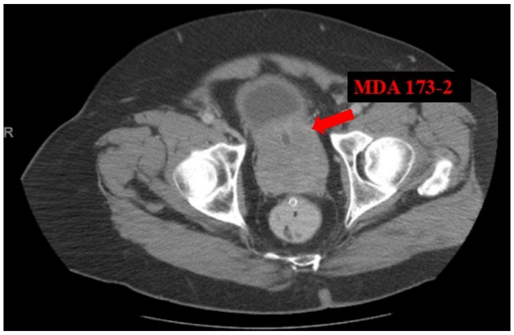

MDA 178-11. The patient donor for MDA 178-11 was 58 years old when he was diagnosed with a PSA 19, Gleason 4+5, cT3bN1M1b de novo metastatic prostate adenocarcinoma in 08/2008. In 09/2008 a biopsy of a sacral lesion confirmed **metastatic prostatic adenocarcinoma positive for PAP**. He was treated with intermittent combined androgen blockade and had a good response but developed recurrent urinary symptoms upon disease progression. Repeat prostate biopsies in 02/2009 revealed **prostatic adenocarcinoma** in all cores. In 08/2009 he underwent a salvage radical prostatectomy which revealed **prostatic adenocarcinoma**. This surgical specimen yielded **MDA 178-11**. He was subsequently treated with diethylstilbestrol-dutasteride, followed abiraterone-dasatinib on clinical trial (to which he had minimal response), docetaxel-carboplatin to which he responded favorably, palliative radiation to sacrum and right hip and cyclophosphamide-vincristine-dexamethasone. In 06/2011 and 11/2011 biopsies of his sacral metastasis continued to reveal **prostatic adenocarcinoma**. In 11/2011 a biopsy of a pelvic lymph node and in 11/2012 a biopsy of a supraclavicular lymph node also revealed **prostatic adenocarcinoma** morphology. In 11/2012 he began treatment with dovitinib on a clinical trial but progressed in 01/2013 and passed away in 03/2013, 4.6 years after his diagnosis.

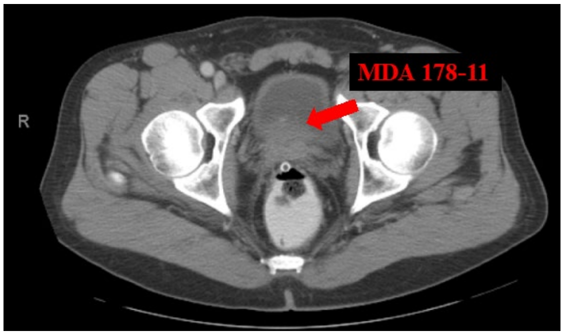

__**MDA 180-30.**__ The patient donor for 180-30 developed lower urinary tract symptoms at the age of 71 and was diagnosed with a PSA 53, Gleason 4+5, cT4N1M1b de novo metastatic prostate adenocarcinoma in 01/2007. He was treated with combined androgen blockade for approximately 2 years followed by docetaxel and carboplatin. In 06/2009 prostate biopsies revealed **high grade prostate adenocarcinoma** involving all cores. He underwent a salvage cystoprostatectomy in 11/2009 with pathology showing **prostatic adenocarcinoma** positive for AR, PSA and synaptophysin but negative for chromogranin A, as previously reported in (3). In 04/2010, he began treatment with diethylstilbestrol and dutasteride but he developed hepatic metastases. In 07/2010 he underwent a biopsy of a liver metastasis that **high grade carcinoma with neuroendocrine differentiation in fibrous tissue** (stains were positive for prostein, PSAP and synaptophysin but negative for chromogranin A). He succumbed to his disease in 12/2010, 4 years after his diagnosis.

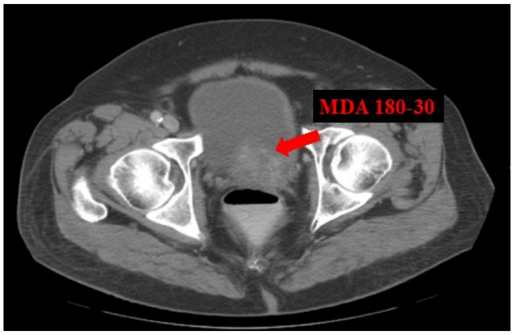

<u>MDA 183-A and MDA 203-A.</u> The patient donor for MDA 183-A and MDA 203-A presented with enlarged inguinal lymph nodes bilaterally and lower urinary tract symptoms and was diagnosed in 01/2010 with a PSA 827, Gleason unknown, cTxN1M1b de novo metastatic prostate carcinoma at age 59. In 01/2010 he underwent a bone marrow biopsy that revealed **metastatic tumor diffusely replacing the medullary space with osteoploriferation and sclerosis that was PSA positive**. This specimen yielded **MDA 183-A**. He began treatment with leuprolide. His PSA nadired at 53ng/dL. In 08/2010 his PSA was 178ng/dL prostate biopsies showed **prostatic adenocarcinoma** with therapy effect. Bicalutamide was added. Diethylstibestrol was prescribed but he did not tolerate it. In 01/2011 a repeat bone marrow biopsy showed **metastatic neoplasm** involving 50% of the medullary space. This specimen yielded **MDA 203-A**. He was then treated with sipuleucel-T followed by radiation to T7-L1 and abiraterone. In 12/2011 he progressed and a bone marrow biopsy showed **metastatic carcinoma** replacing the bone marrow space. He was treated with docetaxel-carboplatin but progressed and passed away in 04/2012, 2.2 years after his diagnosis.

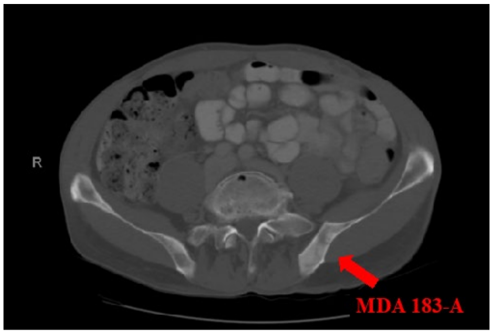

<u>MDA 211-3.</u> The patient donor for MDA 203-A presented with lower urinary tract symptoms and was diagnosed in 02/2011, at the age of 40, with a PSA 4.7, Gleason 4+5, cT4N0M1b de novo metastatic prostate cancer. In 03/2011 rectal wall biopsies revealed **prostate adenocarcinoma.** He began treatment with androgen deprivation therapy but his urinary symptoms worsened so docetaxel-carboplatin was added to his treatment. In 05/2011 an exam under anesthesia revealed a 6x8cm mass and bladder neck biopsy showed **prostatic adenocarcinoma with treatment effect.** In 07/2011 he underwent a radical prostatectomy and partial cystectomy, revealing a prostate gland diffusely replaced by **prostate adenocarcinoma**. This surgical specimen yielded MDA 211-3. He was then treated with sipuleucel-T in 07/2012. In 12/2012 he presented with a nodule at the base of his penis. In 01/2013 a retroperitoneal lymph node biopsy revealed **prostatic adenocarcinoma in fibrous tissue and loose myxoid stroma** and he began treatment with cabazitaxel. In 05/2013 he completed radiation to the penile lesion. In 01/2014 he presented with induration of his penile shaft and nodules on the glans. In 02/2014 a biopsy of these lesions showed **prostatic adenocarcinoma.** He resumed paclitaxel-carboplatin but developed a hypersensitivity reaction to the carboplatin, which had to be substituted for cisplatin. He was subsequently treated with cyclophosphamide-adriamycin-vincristine, a phase I study of crizotinib and dasatinib, radiation to the glans penis and to pelvic bone metastasis, a phase I study of selinexor, a phase I study of listeria vaccine, cabozantinib, ipilimumab-nivolumab and again cabozantinib. In 04/2017 a right iliac bone biopsy revealed prostatic adenocarcinoma. Of note, he was on selinexor for 1 year and responded well to the cabozantinib for several months. Unfortunately, he succumbed to his disease in 12/2018, 8 years after his diagnosis.

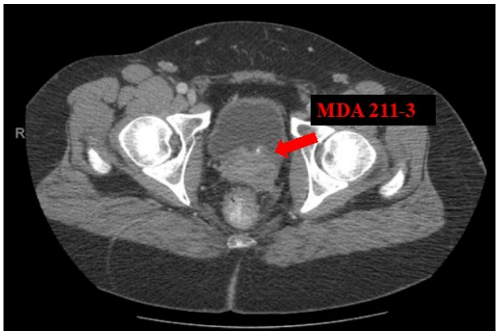

<u>MDA 265-6.</u> The patient donor for MDA 265-6 had presented with lower urinary tract symptoms at age 42 and was diagnosed with a PSA 24, *primary small cell carcinoma*, cT4N1M1b de novo metastatic disease in 04/2012. He was treated with leuprolide but progressed rapidly. In 07/2012 he began etoposide-cisplatin. In 10/2012 a trigone biopsy showed **high grade prostatic adenocarcinoma**. He began abiraterone followed by docetaxel-carboplatin and then, in 04/2013 he underwent a salvage cystoprostatectomy. Pathology showed **high grade prostate adenocarcinoma with neuroendocrine differentiation**, positive for AR, PSA, PAP, chromogranin and synaptophysin. This surgical specimen yielded **MDA 265-6**. He then resumed abiraterone followed by cabazitaxel, to which carboplatin was added after two cycles. In 09/2013 a liver biopsy revealed **metastatic carcinoma** positive for PSA and prostein, weakly positive for synaptophysin and chromogranin A negative. He was then treated with temsirolimus and bevacizumab but ultimately succumbed to his disease in 09/2014, 3.4 years after his diagnosis.

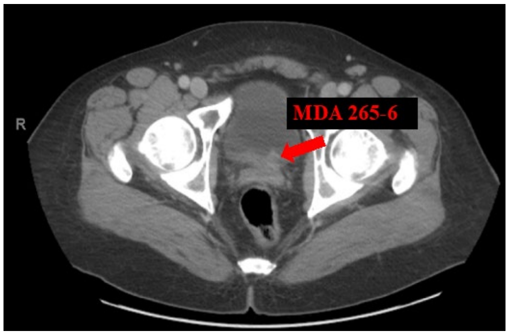

MDA 266-A. The patient donor for MDA 266-A presented with hematuria at the age of 54 and was diagnosed with a PSA 2, Gleason 4+5, cT4N1M0 prostate cancer for which he underwent a radical prostatectomy. His PSA was 0.1 postoperatively and he was treated with goserelin and salvage radiation therapy. In 12/2012 he was found to have peritoneal metastases with ascites and hepatic lesions with bulky soft tissue masses. CEA was 27.6. He underwent paracentesis and ascitic fluid was positive for **metastatic adenocarcinoma**. In 01/2013 a biopsy of a peritoneal nodule also yielded **metastatic adenocarcinoma**. He was treated with 6 cycles of carboplatin and docetaxel followed by cabazitaxel. In 07/2013 a repeat paracentesis again yielded a metastatic adenocarcinoma, negative for PSA. This ascitic fluid specimen yielded **MDA 266-A**. Carboplatin was added to the cabazitaxel but he passed away in 11/2013, 1.5 years after his diagnosis.

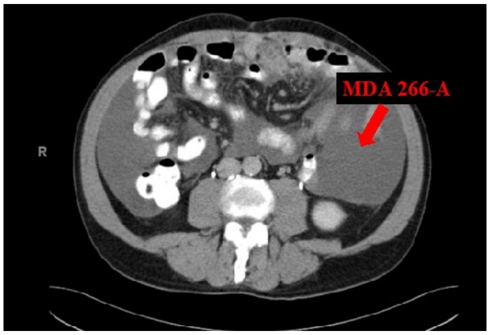

<u>MDA 270-A.</u> The patient donor for 270-A was diagnosed in 08/2013 with a PSA 319, cTxNxM1b de novo metastatic prostate cancer at the age of 62. In 09/2013 he underwent a bone marrow biopsy that revealed **metastatic carcinoma** involving 80% of the marrow space with morphology consistent with prostate primary. He was then started on androgen deprivation therapy with symptomatic and serological response. In 10/2013 he underwent a right nephrectomy for a concurrently diagnosed clear cell carcinoma of the kidney. He was lost to follow up but passed away in 08/2014, 1 year after his diagnosis.

MDA 273-A. The patient donor for 273-A presented with an enlarged supraclavicular node, biopsy proven to be **metastatic poorly differentiated carcinoma** consistent with a prostatic primary, in 07/2009, and was thus diagnosed with a PSA 300, Gleason unknown, cTxN1M1a de novo metastatic prostate cancer at the age of 65. He initiated androgen deprivation therapy and in 05/2010 a repeat biopsy of his supraclavicular lymph node revealed **metastatic adenocarcinoma**, positive for AR and PAP but negative for PSA and prostein. In 03/2012 his PSA rose to 1 in the setting of castrate levels of testosterone and prostate biopsies revealed **high grade prostatic adenocarcinoma** involving all cores. In 08/2012 he underwent a radical prostatectomy which revealed prostatic adenocarcinoma. He remained on leuprolide and in 08/2013 was started on abiraterone but then developed pelvic pain and in 10/2013 was found to have progression in liver and lymph nodes. A retroperitoneal lymph node biopsy revealed **small cell carcinoma**, positive for chromogranin and synaptophysin, negative for PSA. This biopsy specimen yielded **MDA 273-A**. He was treated with cabazitaxel-carbopaltin and cisplatin-etoposide but succumbed to his disease in 06/2014, 4.9 years after his diagnosis.

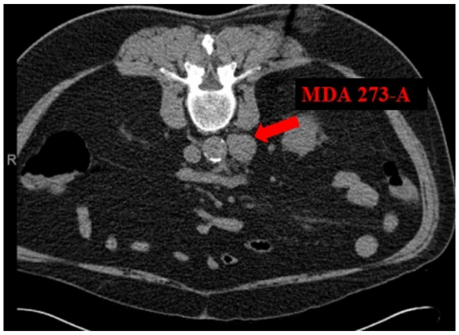

MDA 277-1. The patient donor for 277-1 was diagnosed in 10/2004 with a PSA unknown, Gleason 4+5, cT3aNxM0 prostate cancer at the age of 60. In 01/2005 he underwent a radical prostatectomy and then salvage radiation therapy for a biochemical recurrence. In 2008 he initiated intermittent androgen deprivation therapy but by 05/2012 he had castration resistant disease and began treatment with abiraterone and dasatinib on a clinical trial. In 01/2013 a bone marrow biopsy revealed **metastatic carcinoma** involving 50% of medullary space. In 06/2013 and 10/2013 bone marrow biopsies showed extensive **metastatic carcinoma in a fibrotic marrow**. In 10/2013 he began treatment with cabazitaxel but progressed rapidly and in 12/2013 he initiated radium 223, of which he received 3 cycles. In 02/2014 a right supraclavicular lymph node biopsy revealed **small cell carcinoma positive for synaptophysin and chromogranin and negative for PSA, PAP, and AR.** This specimen yielded **MDA 277-1**. He was treated with etoposide-cisplatin, and then cyclophosphamide-vincristine-diethylstibestrol but passed away in 09/2014, 9.8 years after his diagnosis.

<u>MDA 312-7.</u> The patient donor for 312-7 presented with costovertebral tenderness followed by lower urinary tract symptoms and was diagnosed in 03/2015 with a PSA 2, small cell carcinoma, cT4N1M1c de novo metastatic at the age of 53. Liver and pancreas biopsies confirmed **small cell carcinoma**. He initiated cisplatin and etoposide with ADT and had a good response. In 07/2015 he underwent a cystoprostatectomy revealing a **mixed adenocarcinoma and small cell carcinoma.** This surgical specimen yielded **MDA 312-7**. He subsequently had prophylactic cranial irradiation, another course of cisplatin plus etoposide, and then ipilimumab on clinical trial. He had rapid progression on the latter, was enrolled in hospice and succumbed to his disease in 01/2016, 0.8 years after his diagnosis.

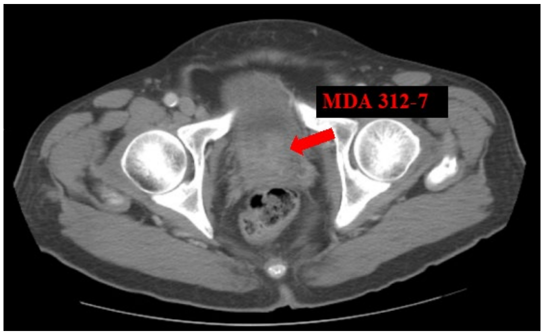

MDA 314-10. The patient donor for MDA 314-10 presented with lower urinary tract symptoms and was diagnosed, in 03/2015 when he was 62 years old, with a PSA 1, small cell carcinoma, cT2cN0M0. He was treated with docetaxel-carboplatin plus ADT with dramatic improvement in disease and then, in 08/2015, underwent a radical prostatectomy that revealed **small cell carcinoma with foci of prostatic adenocarcinoma.** This surgical specimen yielded **MDA 314-10**. He then developed metastatic deposits in the lung, thoracic lymph nodes, liver, and bone for which he received etoposide-cisplatin with interval response. However, he developed metastatic disease in the brain requiring whole brain radiation and then succumbed to his disease in 03/2017, 2 years after his diagnosis.

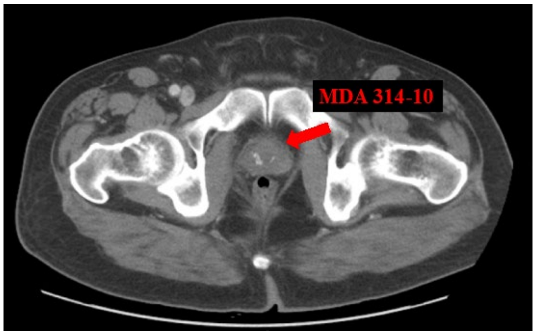

<u>MDA 328-5.</u> The patient donor for 328-5 was 62 years old when he was diagnosed, in 09/2009, with a PSA 12, Gleason 4+5, cT3bN0M0 prostate adenocarcinoma for which he underwent a radical prostatectomy in 12/2009. Postoperatively, he had persistently elevated PSAs and was treated with intermittent leuprolide. In 05/2016 he developed gross hematuria and in 06/2016 a transurethral resection of prostate tumor within the bladder revealed **small cell carcinoma, positive for chromogranin, synaptophysin, CD56 and negative for PSA, PAP, AR.** This resected specimen yielded **MDA 328-5**. He was treated with etoposide-cisplatin, with initial response. In 10/2016, repeat transurethral resection again showed **small cell carcinoma** of the bladder neck and mass was noted to be enlarging and infiltrating the rectum. He was treated with cyclophosphamide-adriamycin-vincristine with initial response. In 03/2017 he was noted to have a single intracranial metastasis for which he underwent Gamma Knife radiosurgery but he passed away in 05/2017, 7.9 years after his diagnosis.

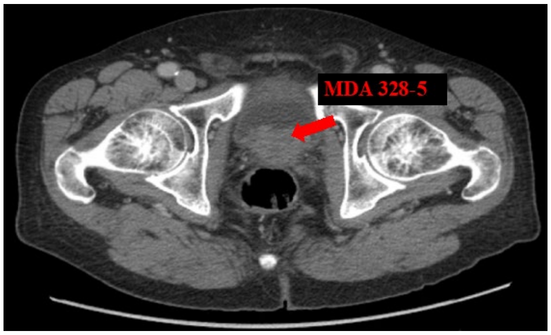

__**MDA 255-A.**__ The patient donor for 255-A had a longstanding history of elevated PSAs and was ultimately diagnosed with a PSA 7.5 (on finasteride), Gleason unknown, cTxNxM1b de novo metastatic prostate cancer in 08/2012 when he was 63 years old and right pelvic and right femoral biopsies, as well as a pelvic lymph node biopsy, revealed p**rostatic adenocarcinoma in the background of extensive necrosis**, PAP and PSA positive. He began treatment with combined androgen blockade but developed worsening urinary symptoms and in 12/2012 underwent a TURP which revealed **small cell carcinoma, PSA and PAP negative.** This specimen yielded MDA 255-A. He was then treated with docetaxel-carboplatin, consolidative radiation to the prostate tumor with concurrent cisplatin, followed by radiation to a brain metastasis, right femur and disease at T11 and S1, and radium-223. Unfortunately, he succumbed to his disease in 02/2014, 1.5 years after his diagnosis.

References:

1. Aparicio A, Tzelepi V, Araujo JC, Guo CC, Liang S, Troncoso P, et al. Neuroendocrine prostate cancer xenografts with large-cell and small-cell features derived from a single patient's tumor: morphological, immunohistochemical, and gene expression profiles. Prostate. 2011;71(8):846-56.
2. Palanisamy N, Yang J, Shepherd PDA, Li-Ning-Tapia EM, Labanca E, Manyam GC, et al. The MD Anderson Prostate Cancer Patient-derived Xenograft Series (MDA PCa PDX) Captures the Molecular Landscape of Prostate Cancer and Facilitates Marker-driven Therapy Development. Clin Cancer Res. 2020.
3. Tzelepi V, Zhang J, Lu JF, Kleb B, Wu G, Wan X, et al. Modeling a lethal prostate cancer variant with small-cell carcinoma features. Clin Cancer Res. 2012;18(3):666-77.
