# Supplementary material for: Genotype-to-Phenotype Associations in the Aggressive Variant Prostate Cancer Molecular Profile (AVPC-m) Components
Source: Cancers (Basel). 2022 Jun 30;14(13):3233. doi: 10.3390/cancers14133233 (PMC9265062; doi:10.3390/cancers14133233)
Supplement: Supplementary file 1 [file cancers-14-03233-s001.zip › Table S1.pdf]

Supplementary Table S1

PDX	Morphology	% malignant cells in FFPE slide	Representative H&E image (all images 20X)
118-B	adenoCa	85	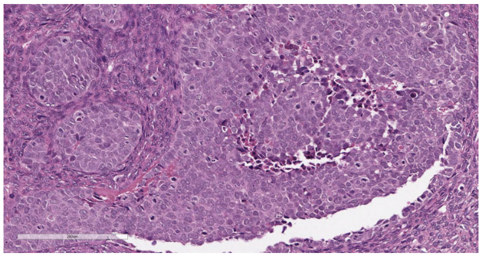
163-A	adenoCa	80	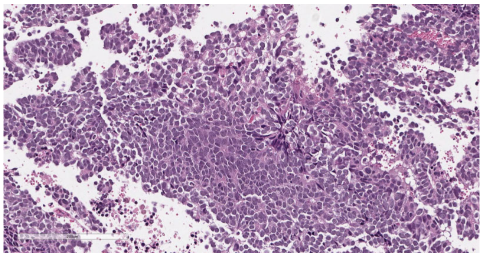
173-2	adenoCa	85	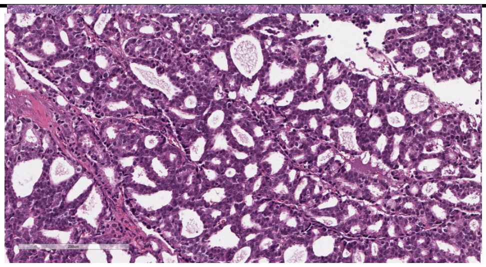
177-B	adenoCa	70	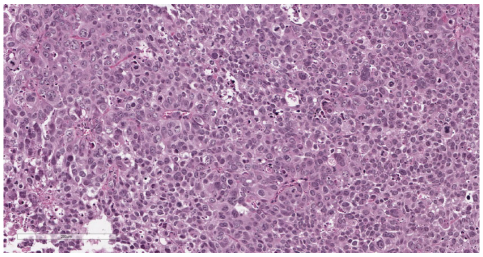
178-11	adenoCa	75	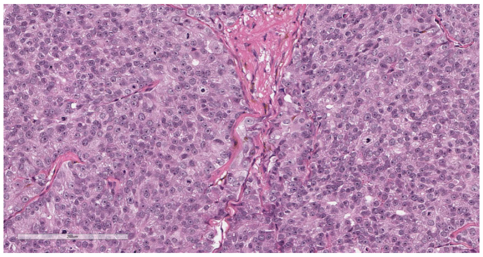

189-1	adenoCa	80	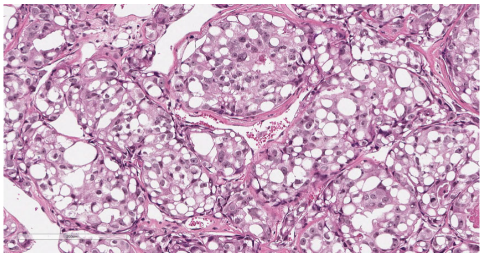
211-3	SCC	75	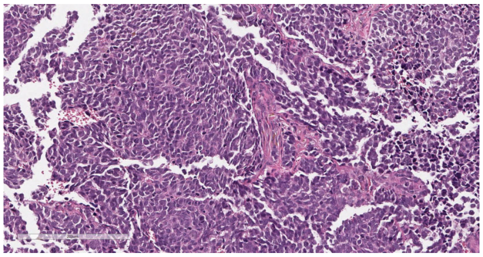
266-A	adenoCa	65	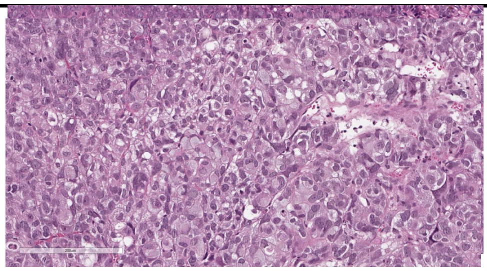
117-9	adenoCa	80	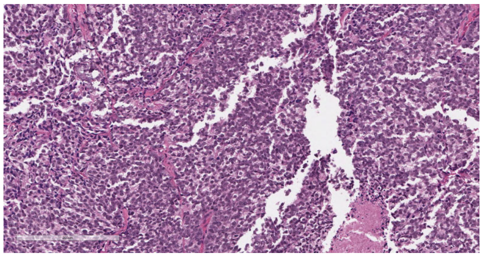
133-4	adenoCa	60	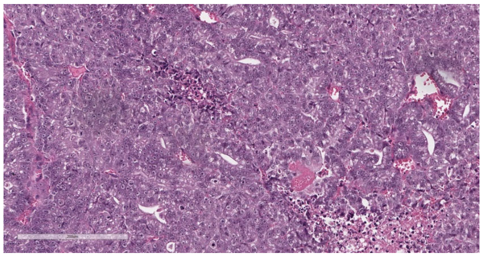
170-1	adenoCa	75	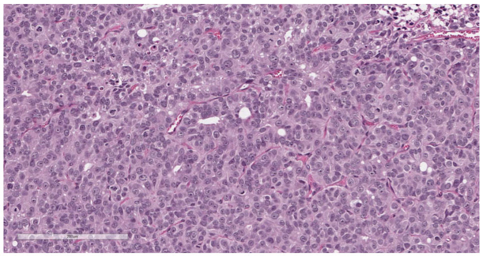

180-30	adenoCa	80	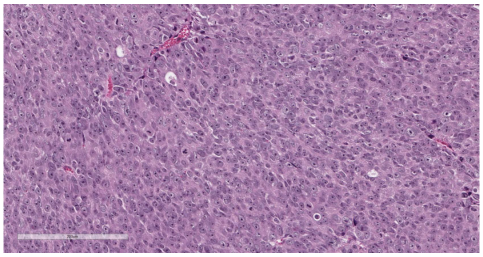
183-A	adenoCa	90	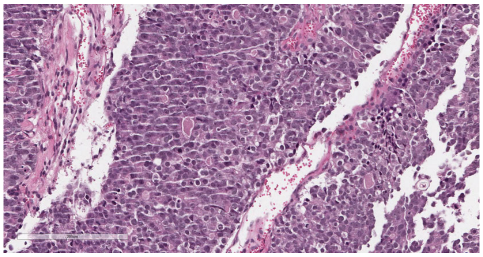
203-A	adenoCa	75	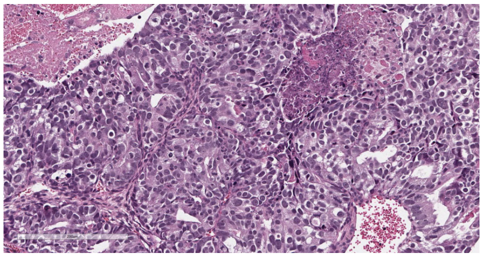
265-6	adenoCa	25	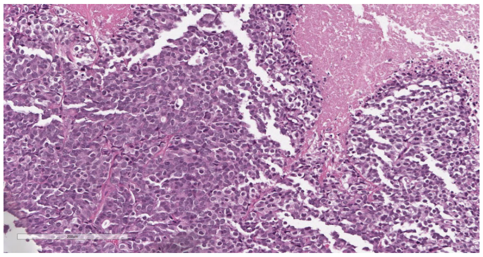
270-A	adenoCa	85	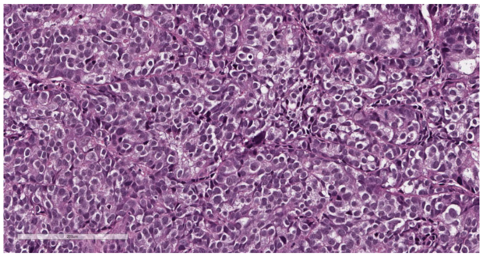
137-8	adenoCa	70	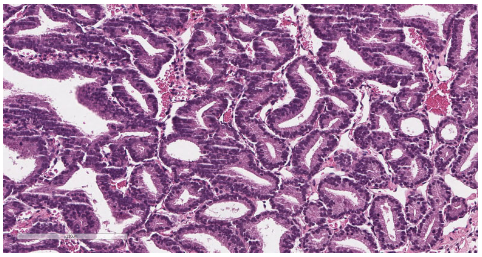

**152-1**	**adenoCa**	**30**	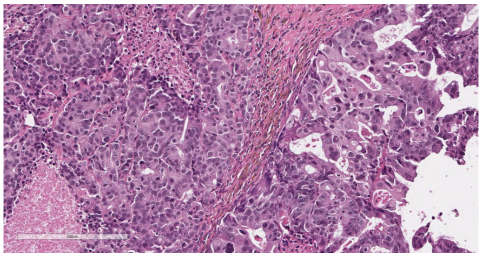
**144-13**	**SCC**	**25**	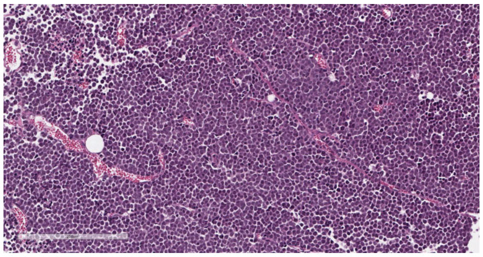
**146-10**	**SCC**	**75**	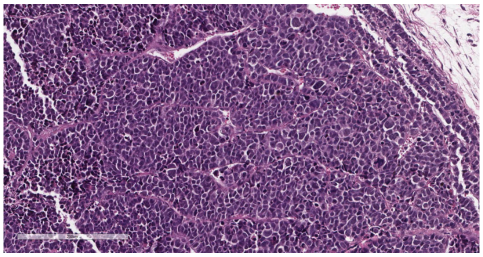
**150-3**	**SCC**	**60**	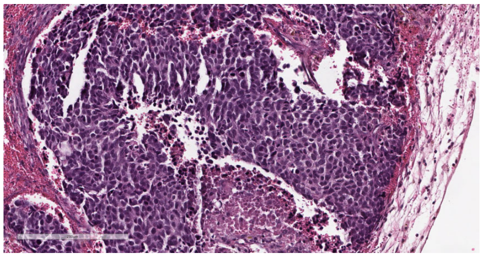
**155-2**	**SCC**	**30**	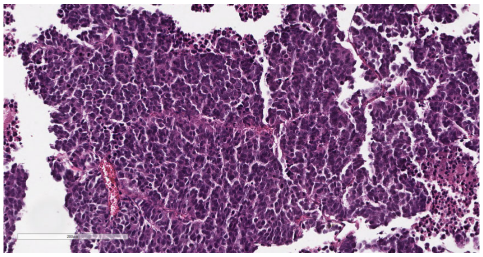
**255-A**	**SCC**	**85**	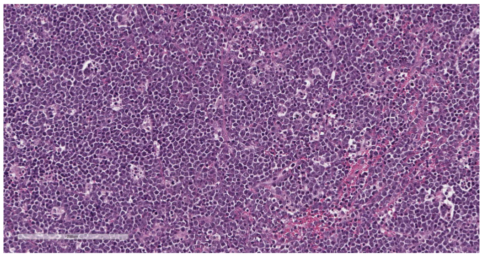

273-A	SCC	50	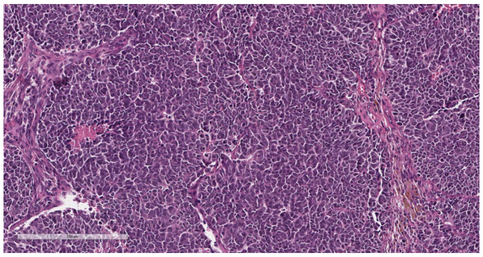
277-1	SCC	75	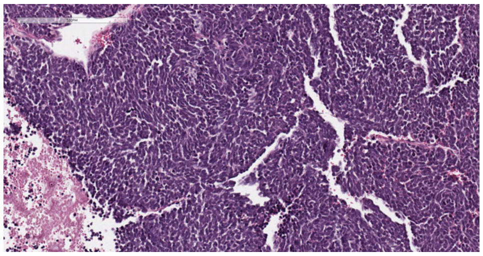
312-7	SCC	60	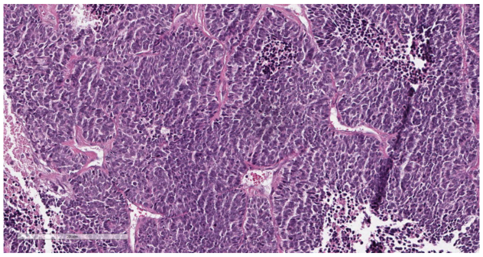
314-10	SCC	90	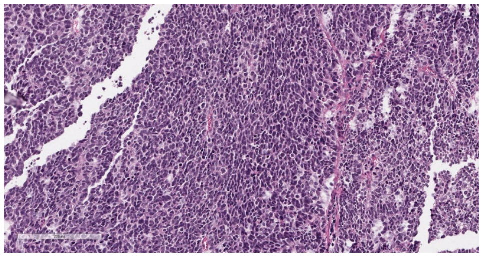
328-5	SCC	50	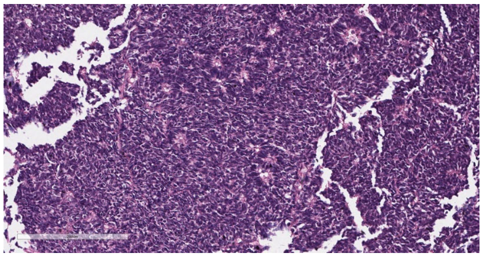
