# Supplementary material for: Genotype-to-Phenotype Associations in the Aggressive Variant Prostate Cancer Molecular Profile (AVPC-m) Components
Source: Cancers (Basel). 2022 Jun 30;14(13):3233. doi: 10.3390/cancers14133233 (PMC9265062; doi:10.3390/cancers14133233)
Supplement: Supplementary file 1 [file cancers-14-03233-s001.zip › Table S2.pdf]

Supplementary Table S2

	Immunohistochemistry: Antibody details								
	LAB 1 (Clinical Lab)			LAB 2			LAB 3		
**Biomarker**	**RB1**	**TP53**	**PTEN**	**RB1**	**TP53**	**PTEN**	**RB1**	**TP53**	**PTEN**
**Vendor**	Calbiochem	Leica Microsystems	Dako	Abcam	Abcam	Cell Signaling Technology	Millipore Sigma	Dako	Biocare Medical
**Cat #**	OP66 (Ab-5)	PA0057	M3627	ab181616	ab32389	9188	OP66 (Ab-5)	M7001	CM278BK
**Clone**	LM95.1	DO-7	6H2.1	EPR17512	E26	D4.3	LM95.1	DO-7	6H2.1
**Type**	Mouse monoclonal	Mouse monoclonal	Mouse monoclonal	Rabbit monoclonal	Rabbit monoclonal	Rabbit monoclonal	Mouse monoclonal	Mouse monoclonal	Mouse monoclonal
**Incubation conditions**	1:30 dilution, 15 min incubation, room temperature	1:1 dilution (ready-to-use), 15 min incubation, room temperature	1:100 dilution, 15 min incubation, room temperature	1:500 dilution, 15 min incubation, room temperature	1: 400 dilution, 15 min incubation, room temperature	1:100 dilution, 15 min incubation, room temperature	1:35 dilution, 60 min incubation, room temperature	1:500 dilution, 60 min incubation, room temperature	1:100 dilution, 60 min incubation, room temperature
**Antigen Retrieval**	Tris EDTA Buffer, pH 9.0, 100°C, 20 min	Tris EDTA Buffer, pH 9.0, 100°C, 20 min	Tris EDTA Buffer, pH 9.0, 100°C, 20 min	BOND Epitope Retrieval Solution 1, pH 6.0, Cat# AR9961, 100°C, 20 min	BOND Epitope Retrieval Solution 1, pH 6.0, Cat# AR9961, 100°C, 20 min	BOND Epitope Retrieval Solution 2, pH 9.0, Cat# AR9640, 100°C, 20 min	Citrate Buffer, pH 6.0, 100°C, 30 min	Citrate Buffer, pH 6.0, 100°C, 30 min	Citrate Buffer, pH 6.0, 100°C, 30 min
**Detection**	HRP-labeled polymer	HRP-labeled polymer	HRP-labeled polymer	HRP-labeled polymer	HRP-labeled polymer	HRP-labeled polymer	HRP-labeled polymer	HRP-labeled polymer	HRP-labeled polymer
**Chromogen Substrate**	DAB	DAB	DAB	DAB	DAB	DAB	DAB	DAB	DAB
**Species Reactivity**	Human	Human	Human	Human, Mouse, Monkey	Human	Human, Mouse, Rat, Monkey and Dog	Human	Human	Human
