# Supplementary material for: Genotype-to-Phenotype Associations in the Aggressive Variant Prostate Cancer Molecular Profile (AVPC-m) Components
Source: Cancers (Basel). 2022 Jun 30;14(13):3233. doi: 10.3390/cancers14133233 (PMC9265062; doi:10.3390/cancers14133233)
Supplement: Supplementary file 1 [file cancers-14-03233-s001.zip › Table S3.pdf]

Supplementary Table S3

	RB1	TP53	PTEN
Negative Control	H526 Cell Line<br><br>Tonsil with Diluent	H522 Cell line<br><br>H841 Cell line with diluent	Cell Signaling Technology Control Slide #8106<br>Placenta with Diluent
Positive Control	H841 Cell Line<br><br>Tonsil	H841 Cell line	Cell Signaling Technology Control Slide #8106<br>Placenta
