# Supplementary material for: Genotype-to-Phenotype Associations in the Aggressive Variant Prostate Cancer Molecular Profile (AVPC-m) Components
Source: Cancers (Basel). 2022 Jun 30;14(13):3233. doi: 10.3390/cancers14133233 (PMC9265062; doi:10.3390/cancers14133233)
Supplement: Supplementary file 1 [file cancers-14-03233-s001.zip › Table S4.pdf]

Supplementary Table S4

	MDA PCa PDX	TP53		RB1		PTEN		AVPC by IHC (Y/N)	AVPC by Genomics (Y/N)	AVPC by IHC and/or Genomics (Y/N)
		Abnormal by IHC (Y/N)	Abnormal by Genomics (Y/N)	Abnormal by IHC (Y/N)	Abnormal by Genomics (Y/N)	Abnormal by IHC (Y/N)	Abnormal by Genomics (Y/N)			
1	177-B	Y	Y (ms Y163N)	Y	Y (del)	Y	N	Y	Y	Y
2	266-A	Y	NA	Y	NA	N	NA	Y	NA	Y
3	144-13	Y	Y (del; ms R273C)	Y	Y (del)	N	Y (del)	Y	Y	Y
4	211-3	Y	NA	Y	NA	N	NA	Y	NA	Y
5	150-3	Y	Y (ms E286K)	Y	Y (del; ss)	Y	Y (del)	Y	Y	Y
6	146-10	Y	Y (del L330fs)	Y	Y (del V450fs)	N	N	Y	Y	Y
7	155-2	Y	Y (ms C275Y)	Y	Y (del)	N	N	Y	Y	Y
8	314-10	Y	Y (ms Y220C)	Y	Y (del)	N	Y (del)	Y	Y	Y
9	273-A	Y	N	Y	Y (del)	N	N	Y	N	Y
10	189-1	Y	NA	N	NA	Y	NA	Y	NA	Y
11	173-2	Y	N	N	N	Y	Y (del)	Y	N	Y
12	117-9	Y	N	N	N	N	N	N	N	N
13	328-5	Y	N	Y	Y (del; ns Q631X)	N	N	Y	N	Y
14	178-11	Y	N	N	Y (del)	Y	Y (del)	Y	Y	Y
15	180-30	Y	Y (ms R175L)	N	Y (ms I470T)	N	N	N	Y	Y
16	312-7	Y	Y (ns E258X)	Y	Y (del)	N	N	Y	Y	Y
17	137-8	Y	NA	N	NA	Y	NA	Y	NA	Y
18	203-A	N	N	N	N	Y	Y (del)	N	N	N
19	118-B	N	NA	Y	NA	N	NA	N	NA	N
20	163-A	N	Y (ns R80X)	Y	Y (del)	N	N	N	Y	Y
21	133-4	N	Y (del P62fs)	N	N	Y	Y (del)	N	Y	Y
22	170-1	N	Y (del)	N	N	N	Y (del)	N	Y	Y
23	183-A	N	N	N	N	Y	Y (del)	N	N	N
24	265-6	N	Y (ns R213X)	N	Y (del)	N	N	N	Y	Y
25	270-A	N	NA	N	NA	N	NA	N	NA	N
26	152-1	N	NA	N	NA	Y	NA	N	NA	N
27	255-A	N	NA	Y	NA	Y	NA	Y	NA	Y
28	277-1	N	Y (ns W68X; ss)	N	Y (del)	N	N	N	Y	Y

NA: Not Available

AVPC

non-AVPC
117-9, 203-A, 183-A, 270-A, 152-1, 118-B

ms = missense mutation
ns = nonsense mutation
ss = splice site mutation
fs = frame shift mutation
