# Supplementary figures and images for: Genotype-to-Phenotype Associations in the Aggressive Variant Prostate Cancer Molecular Profile (AVPC-m) Components
Source: Cancers (Basel). 2022 Jun 30;14(13):3233. doi: 10.3390/cancers14133233 (PMC9265062; doi:10.3390/cancers14133233)

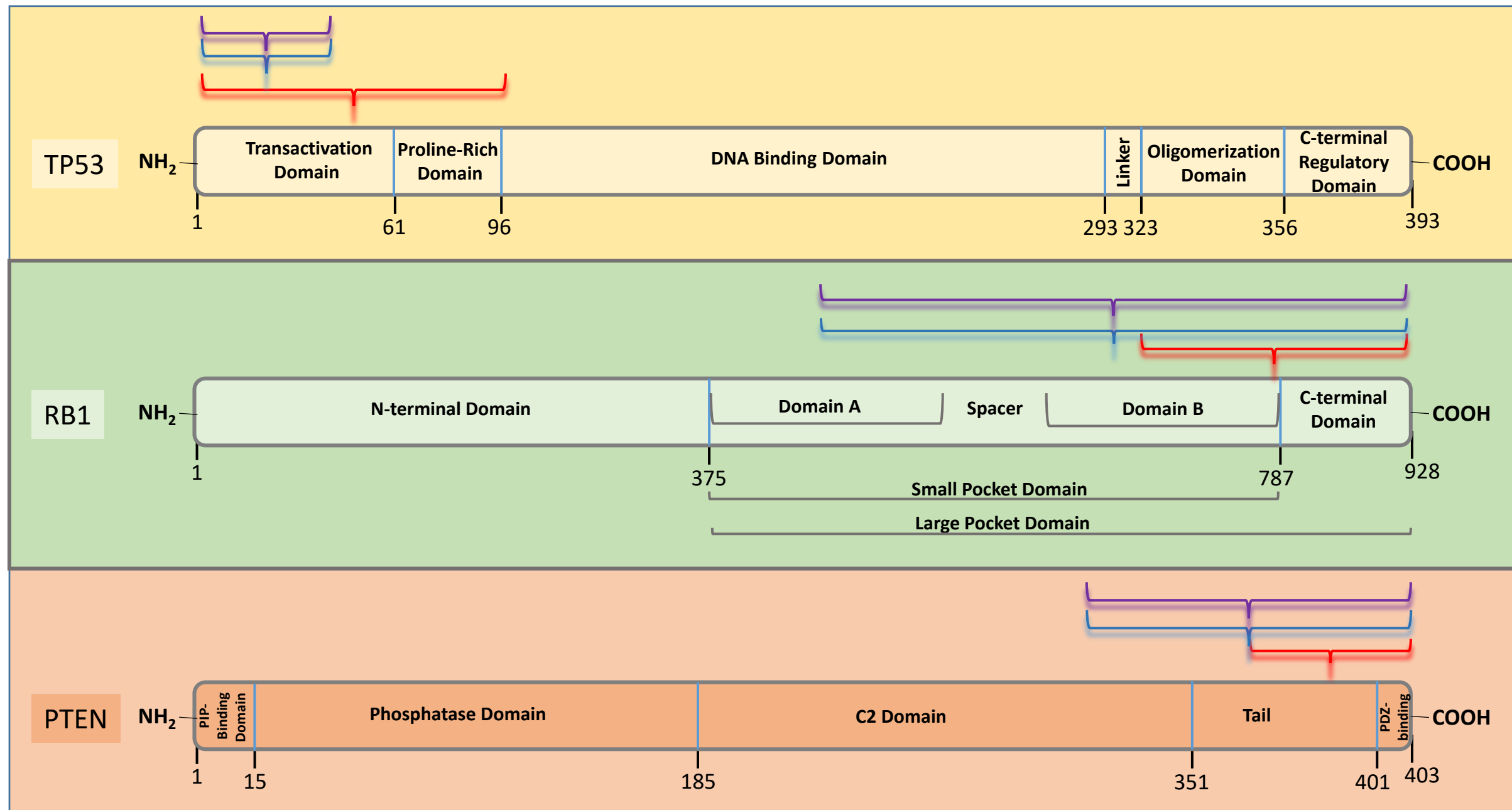

Supplementary Figure S1

LAB 1 (Clinical Lab)  
LAB 2  
LAB 3

Supplement: Supplementary file 1 [file cancers-14-03233-s001.zip › Figure S1.pdf]

Supplementary Figure S2

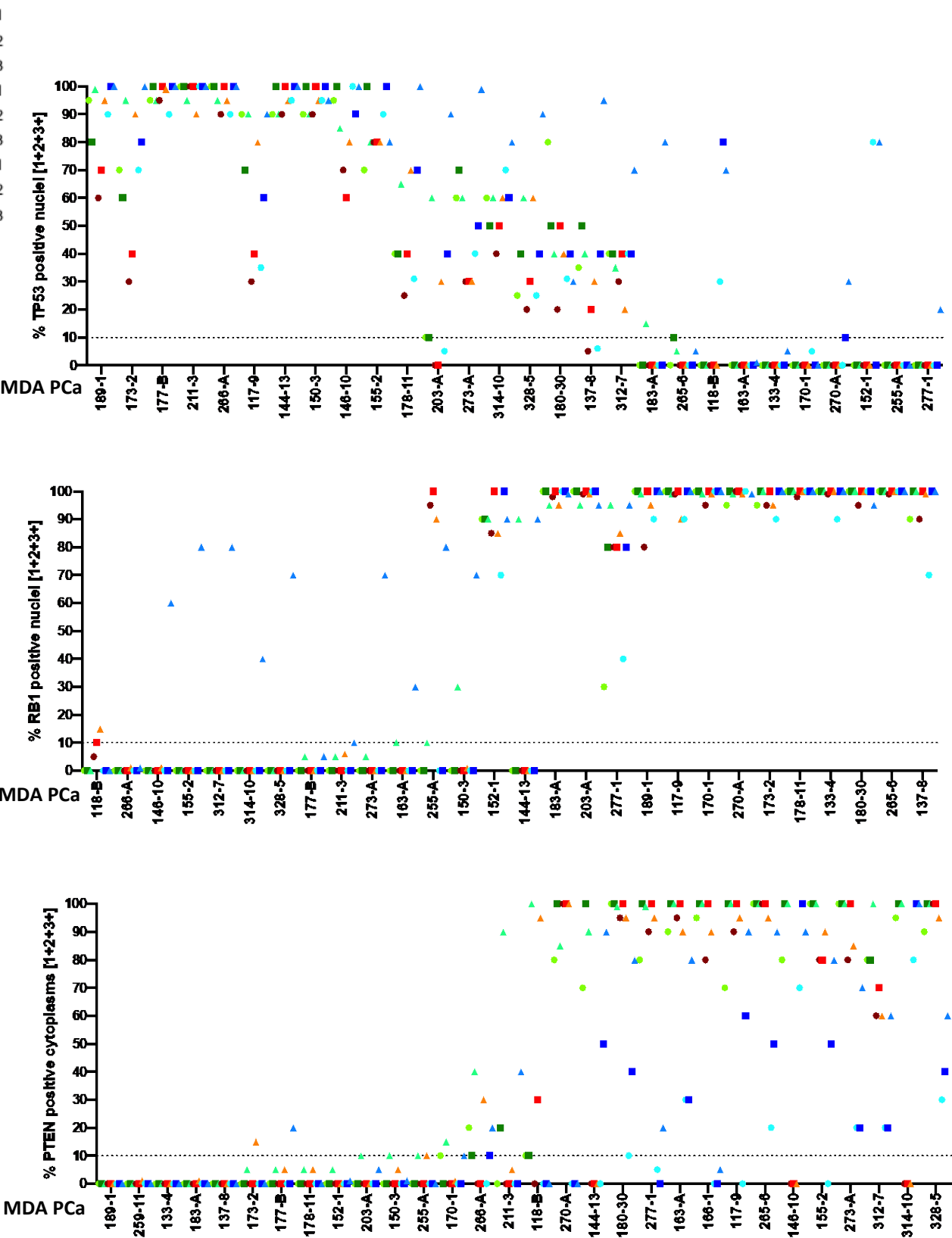

Supplement: Supplementary file 1 [file cancers-14-03233-s001.zip › Figure S2.pdf]

Supplementary Figure S4-1

TP53

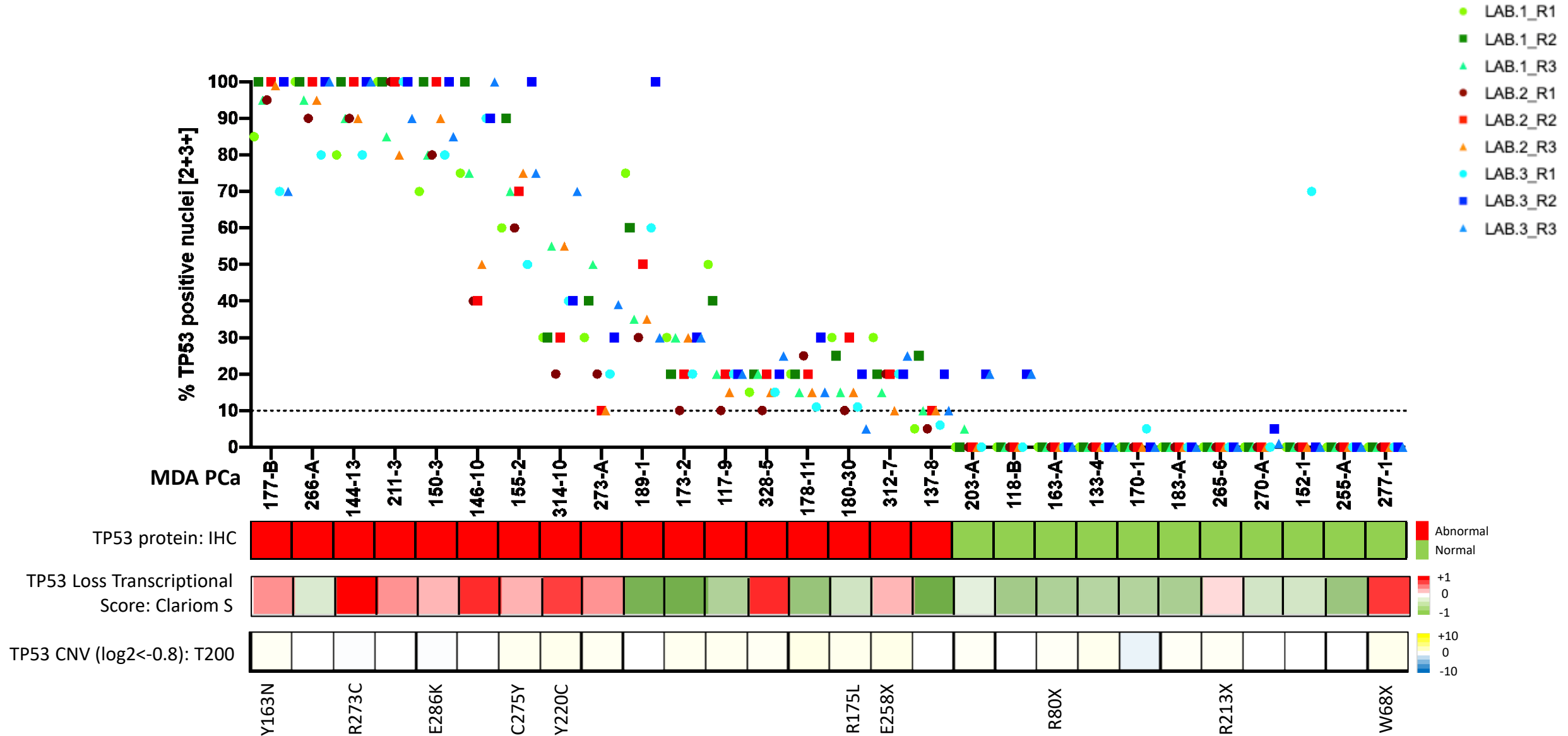

# Supplementary Figure S4-2

RB1

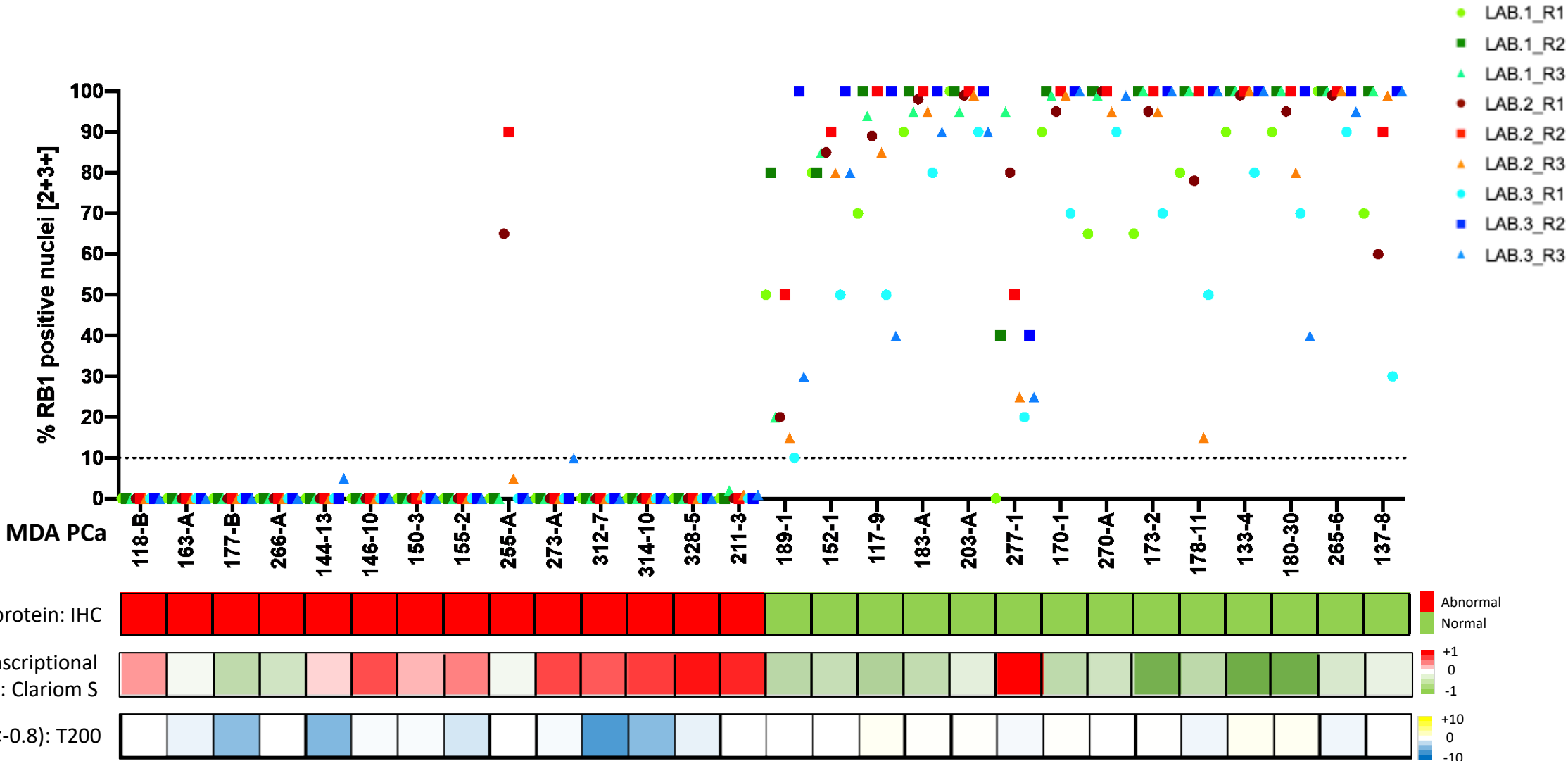

# Supplementary Figure S4-3

## PTEN

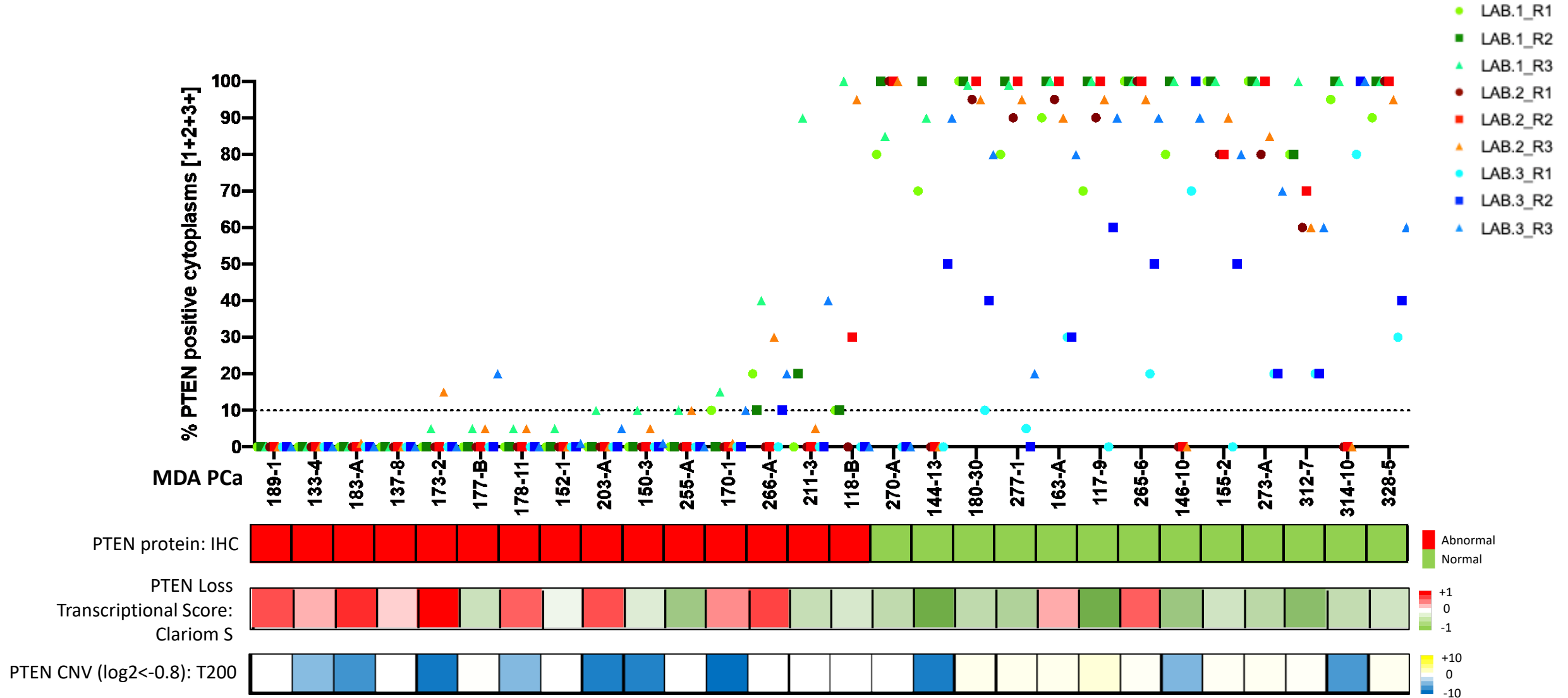

Supplement: Supplementary file 1 [file cancers-14-03233-s001.zip › Figure S4.pdf]
